# Modern Trends in Plant Genome Editing: An Inclusive Review of the CRISPR/Cas9 Toolbox

**DOI:** 10.3390/ijms20164045

**Published:** 2019-08-19

**Authors:** Ali Razzaq, Fozia Saleem, Mehak Kanwal, Ghulam Mustafa, Sumaira Yousaf, Hafiz Muhammad Imran Arshad, Muhammad Khalid Hameed, Muhammad Sarwar Khan, Faiz Ahmad Joyia

**Affiliations:** 1 Centre of Agricultural Biochemistry and Biotechnology (CABB), University of Agriculture, Faisalabad 38040, Pakistan; 2 Nuclear Institute for Agriculture and Biology (NIAB), P.O. Box 128, Faisalabad 38000, Pakistan; 3 School of Agriculture and Biology, Shanghai Jiao Tong University, Shanghai 200240, China

**Keywords:** CRISPR/Cas9, genome editing, plant breeding, multiplex genome editing, crop improvement, TALEN, ZFN, biotic stress, abiotic stress

## Abstract

Increasing agricultural productivity via modern breeding strategies is of prime interest to attain global food security. An array of biotic and abiotic stressors affect productivity as well as the quality of crop plants, and it is a primary need to develop crops with improved adaptability, high productivity, and resilience against these biotic/abiotic stressors. Conventional approaches to genetic engineering involve tedious procedures. State-of-the-art OMICS approaches reinforced with next-generation sequencing and the latest developments in genome editing tools have paved the way for targeted mutagenesis, opening new horizons for precise genome engineering. Various genome editing tools such as transcription activator-like effector nucleases (TALENs), zinc-finger nucleases (ZFNs), and meganucleases (MNs) have enabled plant scientists to manipulate desired genes in crop plants. However, these approaches are expensive and laborious involving complex procedures for successful editing. Conversely, CRISPR/Cas9 is an entrancing, easy-to-design, cost-effective, and versatile tool for precise and efficient plant genome editing. In recent years, the CRISPR/Cas9 system has emerged as a powerful tool for targeted mutagenesis, including single base substitution, multiplex gene editing, gene knockouts, and regulation of gene transcription in plants. Thus, CRISPR/Cas9-based genome editing has demonstrated great potential for crop improvement but regulation of genome-edited crops is still in its infancy. Here, we extensively reviewed the availability of CRISPR/Cas9 genome editing tools for plant biotechnologists to target desired genes and its vast applications in crop breeding research.

## 1. Introduction

Food security is the most crucial challenge in the current scenario of a rapidly growing global population. According to cautious estimates, the global population will escalate to ten billion by the end of 2050 and a 60–100% rise in global food production will be necessary [1]. Besides extreme weather, increasing biotic and abiotic stressors, a growing population, and shrinking availability of agricultural land and water resources are important constraints for food production and farming. Over the past few decades, improvements in crop plants have contributed by deciphering numerous biological mechanisms and elucidating the role of genetic and epigenetics factors [2]. Crop breeders and plant scientist are striving hard to understand mainly the genetic mechanism underlying unique plant responses towards environmental stressors. Recently, numerous novel genes and their regulatory pathways have been identified in plants [3,4]. For crop improvement and development of elite cultivars with increased productivity, a breeding strategy of “cross the elite with the elite and wait for the best” has been applied, concentrating on the genes linked with vital agronomic traits [5]. Classical plant breeding strategies for crop improvement are more challenging which take a long time for germplasm selection. On the other hand, modern tools for genome editing (GE) exhibit the capability of integrating a foreign gene into a predetermined site of the genome precisely, allowing for accurate substitution of an existing allele with an alternative one [6,7]. Genome editing has emerged as a tremendous strategy for efficient and targeted genome manipulations, especially for crops which have complex genomes and which are difficult to improve through conventional breeding approaches [6].

For basic as well as applied plant biology, the unstable and non-specific transgene incorporation in the host genome has been a matter of concern for edible crop species [8]. The discovery of programmed sequence-specific nucleases (SSNs) has facilitated precise gene editing. In both plant and animal systems, application of SSNs for accurate GE has been recognized as a breakthrough in genome engineering. The SSNs can be applied to produce several kinds of mutations, such as insertions, deletions, replacement, substitutions, integration of specific sequence of DNA at a desired locus, and site-directed substitutions across many organisms and cell types. Though all types of SSNs have unique features, the mechanism for producing double-strand breaks (DSBs) in the target DNA is similar for all. The DSBs created by SSNs are reconstructed via non-homologous end joining (NHEJ) or homology-directed recombination (HDR). Non-homologous end joining is an error-prone DNA repair mechanism that facilitates direct end-joining of DSBs without involving a homologous template and can generate insertions or deletions at target sites to develop gene knockouts. Additionally, NHEJ can also be applied to introduce insertions at the point of the DSB during operation of the repair mechanism. On the other hand, the HDR repair pathway is a highly accurate mechanism that needs a homologous template to mediate repair and can be used to attain precise changes like gene insertion and gene replacement [6,9,10]. As compared to transgenic strategies, which result in inadvertent gene insertions and sometimes random phenotypical characters, GE approaches produce well-defined mutants, proving GE as a powerful technique for plant breeding and functional genomics. In contrast to transgenic plants, genome-edited plants have the added benefit of site specificity [11]. In breeding programs, these improved plants can be proven useful and subsequent species can be employed reliably with less concerns and comparatively minor monitoring methods are needed in contrast to traditional genetically engineered plants [12]. 

## 2. Modern Trends in Plant Genome Editing

In recent years, many fascinating GE approaches have been established because of the advancements in molecular biology, which have permitted site-specific and accurate editing in many genomes [8]. In GE, engineered nucleases are composed of a sequence-specific DNA binding domain merged with a non-specific nucleases domain. Targeted genes can be precisely cleaved by such fused nucleases and nicks can be repaired with the help of HDR or NHEJ [13,14]. A vital strategy to execute targeted GE through SSNs is to generate DSBs at targeted sites; these nicks prompt the activation of the DNA repair mechanism through the HDR or NHEJ pathway [15]. The DNA repair system of the HDR pathways requires a homologous template to repair the DSB, whereas the two ends of DSBs are directly ligated in the NHEJ pathway [15]. Though NHEJ is more common, there are some flaws which make it undesirable in many studies. The major disadvantage of this process is that it produces insertions or deletions of different sizes during the repair mechanism, which may produce off-targets. In contrast to NHEJ, the repair mechanism via HDR is more accurate and reliable, which depends on homologous DNA to repair the DSB [16]. Thus, SSNs can be applied to manipulate the genomic sequences by targeted addition or deletion of specific nucleotides in the targeted locus [10].

Recently, great achievements have been made in the era of genome engineering with the development of meganulceases (MNs), zinc-finger nucleases (ZFNs), transcription activator-like effector nucleases (TALENs), and clustered regularly interspaced short palindromic repeats/ CRISPR-associated protein 9 (CRISPR/Cas9). Experimental proofs gradually showed that these SSNs were not only used for gene insertion or inactivation, but also significantly enhanced the effectiveness of homologous recombination and, thus, allowed more precise gene replacement events. In 1993, Puchta and co-workers [17] provided the first evidence of homologous recombination in plant cells by using SSNs. After the discovery of ZFNs in 1996 by Kim and colleagues [18], extensive efforts have been made for progressive advancement with this tool, which offered a significant breakthrough in plant GE. In 2003, scientists were able to inactivate genes using ZFNs for the first time [19]. In 2005, the first SSN-based mutagenesis via ZFNs was carried out successfully in plants [20]. Therefore, ZFNs have the ability to produce site-specific DSBs and have many applications in genome engineering [21]. Later TALENs were included to the toolbox of SSNs for programmed genome engineering [22]. Genome manipulation through engineered nucleases had gained much importance by the end of 2011, and *Nature Methods* crowned it as the “Method of the Year”. In 2012, *Science* chose it as the “Breakthrough of the Year” due to the significant progress achieved in GE using TALENs. Recently, an emerging GE nuclease, “CRISPR/Cas9”, was added to the toolbox for editing nucleases. In 2013, the first CRISPR/Cas9-based GE event was reported in eukaryotes [23]. In 2015, Ma’s group [24] developed the multiplex genome editing mechanism in monocots and dicots. In 2013 and 2015, it was selected by *Science* as the “Breakthrough of the Year”. Furthermore, advancements in the CRISPR/Cas system introduced a more precise technique of base editing, which was heralded again by *Science* in 2017 as the “Breakthrough of the Year”. CRISPR/Cas9-based GE has tremendously revolutionized genome engineering since the initial few research articles were published in *Nature Biotechnology* [25,26]. All the above mentioned approaches have been extensively employed for GE and caused mutations via site-directed substitutions, replacement, deletions, and insertions at specific sites in the genome [10].

**Figure 1:**
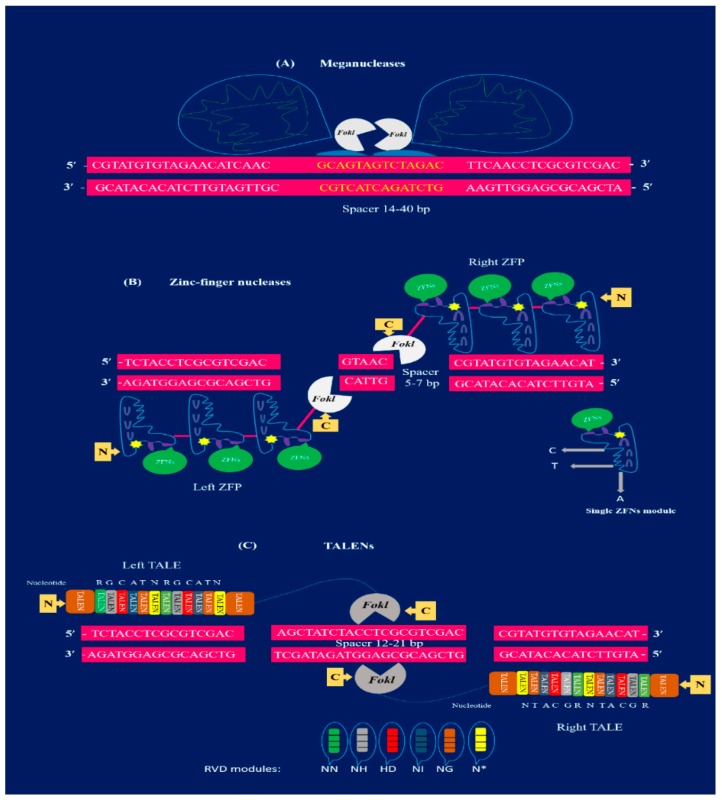
Structural illustration of first-generation genome editing tools: (**A**) meganucleases (MNs) have multifunctional domains with the ability to bind double-stranded target DNA and generate DSBs. The meganuclease is demonstrated to bind a target spacer sequence of 14–40 bp (yellow). The FokI nuclease cuts the target sequence (color). (**B**) Representation of ZFN bound to the target sequence of 18–36 bp long. Each monomer of ZFN (blue) is made by ZFP. There are two basic domains: the DNA binding domain at N-terminus and the catalytic domain with FokI nuclease (white) present at the C-terminus. The connection among these domains is indicated with a pink line. The ZFN modules are merged with Fok1 (white) and dimerized to cut the target sequence at a spacer 5–7 bp (pink) to produce DSBs. (**C**) Two TALEN dimers bound to the target sequence (pink) site. Each module of TALENs are composed of TALE that contain 33–35 amino acid repeats. The pair of TALENs are separated by a spacer region of 12–21 bp (pink). There are specific RVD modules (green NN, grey NH, red HD, dark blue NI, orange NG, and yellow N) that can recognize only one single nucleotide. TALE modules are dimerized to fuse with FokI (at C-terminus) to produce DSBs in the spacer region.

**Figure 2:**
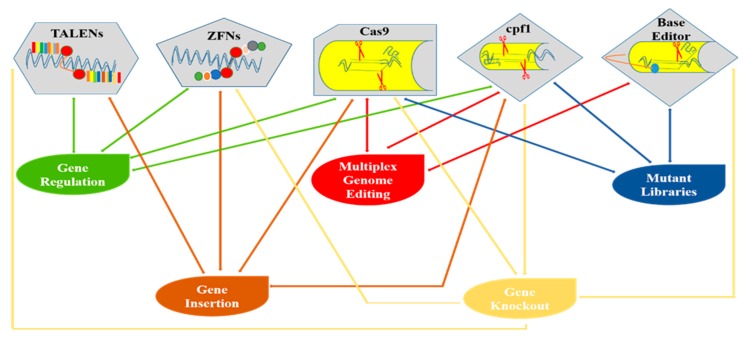
Diagrammatical representation of various genome editing tools and their applications in plants.

**Figure 3:**
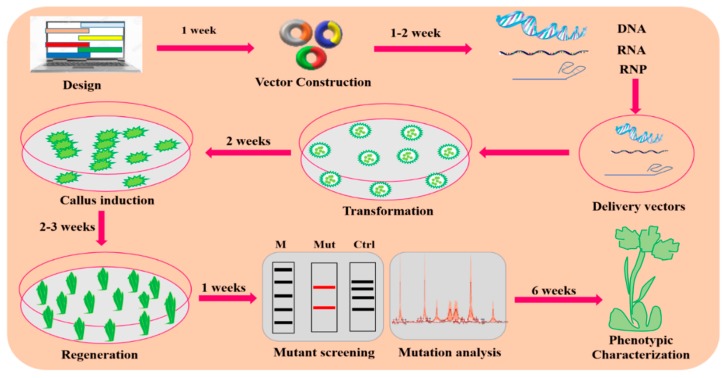
A general description of the GE mechanism in plants. Plant GE typically consists of the following steps: designing and construction of vectors, targeted delivery of vectors via *Agrobacterium*-mediated transformation or biolistic for transformation, callus induction and regeneration, mutation screening and analysis, and phenotypic characterization for the desired trait.

TALENs, ZFNs, and MNs are the first-generation editing tools for genome manipulation as, illustrated in Figure 1. However, they are time consuming and require lengthy protocols to attain target specificity. As compared to first-generation GE approaches, second-generation GE tools such as the CRISPR-Cas9 technique are easier to design, cost effective, and robust [26,27,28]. The CRISPR/Cas9 toolkit is very simple to design, as it involves only single-guided RNA (sgRNA) and the Cas9 protein in contrast to TALENs and ZFNs. Additionally, the procedure involved in TALENs and ZFNs are complex because they require protein engineering for their construction. Due to the presence of these constraints, applications of TALENs and ZFNs in plants have been limited [9]. Continuous innovation for efficient GE has expanded the applications of the CRISPR/Cas9 system in several fields of plant science and is quickly becoming a highly promising GE tool [6,11,23,24,25,26]. The plant GE tools and their corresponding applications are depicted in Figure 2, while successive steps involving the GE strategies are shown in Figure 3.

In the present review, we discuss fascinating GE tools for crop improvement. We briefly describe first-generation genome editing tools such as TALENs, ZFNs, and MNs and comprehensively elaborate on second-generation genome editing strategies with special focus on the applications of the CRISPR/Cas9 system in plant breeding for crop improvement. We briefly outline historical background, structural organization, and mode of action of the CRISPR/Cas9 toolbox. We describe the workflow of CRISPR/Cas9 from vector design to mutant screening. We also highlight recent breakthrough events in technology improvement in the CRISPR/Cas9 system. Furthermore, we discuss the recent role of CRISPR/Cas9 technology in crop breeding to develop the best performing cultivars with biotic and abiotic stress resilience, improving yield-related traits and production of high-quality crops. Finally, we outline the future outlook of CRISPR/Cas9 and pinpoint the current challenges with respect to the regulation of edited crops and their safe use. 

### 2.1. Meganulceases

In SSNs, MNs were the pioneering class of nucleases (Figure 1A), extensively applied for plant GE [29,30]. Meganulceases were also termed as homing endonucleases. Later, they were utilized for generating DSBs in several genomes [31]. Meganulceases have the ability to recognize target DNA sequences of about ~12–40 bp, that make MNs the most efficient delivery approach for all vectors including plant RNA viruses [32]. As compared to different SSNs, MNs are difficult to re-design for target sequences different than their natural ones. Non-modular properties of the specific proteins are the main reason for hindrance in re-designing MNs. So, for plants, the applications of MNs have been restricted to only naturally existing MNs such as I-SceI and I-CreI nucleases [29].

### 2.2. Zinc-Finger Nucleases

In plants, ZFNs are extensively being applied for plant GE [33]. Zinc-finger nucleases are one of the main techniques for genome manipulations which are very beneficial in various GE applications. Zinc-finger nucleases have been widely used for target specific mutagenesis to disrupt the gene function and produce several gene knockouts [34]. GE with ZFNs has demonstrated the production of herbicide-resistant plants, and various kinds of targeted and specific gene insertion have also been unveiled [35]. In plant biotechnology, zinc-finger proteins (ZFPs) can be exploited in two ways: ZFNs and ZFN-TFs. Due to the flexible nature of ZFPs, it provides a striking basis for modeling ZFNs with desired sequence-specific domains to produce DSBs and facilitate GE [36,37]. In 1996, ZFNs were reported for the first time and named as chimeric restriction enzymes. According to this research, chimeric restriction enzymes were developed by associating the non-specific FokI with the DNA binding domain of two dissimilar ZFPs. The ZFNs were constructed by the fusion of chimeric proteins that were composed of DNA cleavage and a DNA binding domain. A set of 3–6 Cys2His 2 ZFs constructed the DNA binding domain, while a Fok1 restriction enzyme was generated by the DNA cleavage domain [18]. FokI is homodimeric in nature and belongs to the type IIS class of restriction enzymes isolated from *Flavobacterium okeanokoites* [18]. The domain of FokI nuclease needs to dimerize in order to cut DNA [38]. Two ZFN monomers are required having FokI dimerization and C-terminal fusion for active cleavage when binding to DNA. Target DNA with 9–18 bp has been recognized by each monomer containing 3–6 ZFs (Figure 1B). Consequently, each monomer of ZFN targets the spacer region of 5–7 bp located in the adjacent half-site and dimerize to perform the cleavage activity for targeted DNA [10]. ZFNs, as compared to MNs, are small in size (about 300 aa in one monomer and 600 aa in a pair of nucleases), enabling them responsive to many delivery procedures. In the last two decades, ZFNs have been applied for site-specific mutations in plants such as *Arabidopsis thaliana*, soybean, maize, tobacco, and petunia [36,39,40]. However, modular association of ZFs has gained partial achievement [41]. Currently, ZFNs are not recommended in several cases due to the lower target specificity, limited amount of specific target domains, and large number of non-targeted editing [42].

### 2.3. TALENs

Another interesting tool for GE is termed as transcription activator-like effector nucleases (TALENs). These are modern inclusions to the SSNs resources and have been extensively applied for GE in plants [43]. In 1989, TALENs were first discovered when a pathogenic bacterium called *Xanthomonas* was studied for many plant varieties [44]. *Xanthomonas* is responsible for uncontrolled growth of plant cells due to the synthesis of a novel protein termed as transcription activator-like effectors (TALEs) that target specific DNA sequences and greatly influence gene expression [45]. For targeted GE, TALENs are manipulated by changing the TALE repeated domains required for specific target identification and are successively linked to Fok1 nuclease to obtain suitable TALEN. The TALENs that recognize 12–21 bp extend, likewise to ZFNs, and require a spacer region of 14–20 bp for Fok1 dimerization with a pair of TALENs (Figure 1C) [10].

TALENs have the advantage over other SSNs such as MNs and ZFNs because of their modular domain. The domain for TALENs contains 33–35 aa in direct sequence repeats and two amino acids are called repeat variable di-residues (RVDs) in these repeats. The RVDs are responsible for recognition of specific nucleotides which includes thymine; NI, HD, cytosine; NG and adenine; and NN. A single RVD associated with every single nucleotide in combined mechanisms was identified that has the ability to design specific DNA binding motifs and remove the remodeling issues faced in the case of ZFNs and MNs [22,46,47,48]. Target specificity is another advantage of TALENs over other nucleases. Typically, 15–20 RVDs are used in order to design TALEN monomers with more than a 30 bp target site. As compared to ZFNs, TALENs reduce the toxicity and are more specific having large target sites [49]. A large size of about ~950 aa to ~1900 aa is the only drawback of TALENs for use as an accurate tool for GE. TALENs are usually carried to cells via direct integration of DNA or by integration of a construct-harboring TALEN-encoding unit into the genome. TALENs have been successfully applied in plants such as rice [43], *Arabidopsis* [22], tobacco [42], and *Brachypodium* [26] for GE. TALENs are more extensively exploited for targeted GE as compared to ZFNs, but they still need an efficient way to assemble tandem repeats for binding to the targeted DNA region. Furthermore, the repetitive nature and large size pose as big hurdles for the successful delivery of TALENs [45]. Some reported events of MN-, TALEN-, and ZFN-mediated mutagenesis in plants are described in Table 1.

## 3. CRISPR/Cas9 System

Recently, a fascinating GE tool CRISPR/Cas9 was identified for targeted genome manipulations and to express desired genes in numerous organisms [6,65]. The CRISPR/Cas9 system has emerged as the most powerful tool for GE in many species including plants [26]. The latest ground-breaking technology of CRISPR/Cas9 is basically present as an adaptive immune system of type II prokaryotes and protects them against invading organisms during phage infection by spacer acquisition, biogenesis, and target degradation [9]. The toolbox of CRISPR/Cas9 was adapted from bacteria as well as Archaea and in included in the toolbox of engineered nucleases [23,66]. There are two main components of the CRISPR/Cas9 system: a single guide RNA (sgRNA) that identifies a specific DNA sequence and the Cas9 protein which produces DSBs at a targeted site [9]. Therefore, when changing the design of sgRNA, numerous desired sites can be targeted, which makes it simpler to handle than TALENs and ZFNs [10]. 

### 3.1. Discovery of CRISPR/Cas9 Wonder

The discovery of the CRISPR/Cas9 system dates back to 1987 when Ishino and his colleagues first identified CRISPR while studying the *iap* gene in the genome of *E. coli*. During the cloning of the *iap* gene, they unexpectedly cloned a specific portion of CRISPR, and at the conclusion of their experiment, revealed that the bacterial genome consisted of a successive array of repeats [67]. After this discovery, an Archaea (*Haloferax mediteranii*) was also found to contain the CRISPR sequences [68]. Mojica et al. (2000) reported a similar type of regularly spaced repeats in *Haloferax mediterranei* and *Haloferax volcanii*, having interrelated functions [69]. Only prokaryotes were considered to have such repetitive sequences, which were named as CRISPR but were not present in eukaryotes and viruses [70]. These short repeats have an average length of 32 bp but are of different sizes from 21 to 47 bp in different organisms. Every repeat has a unique sequence of nucleotides that are extremely conserved in specific species [71]. It was unveiled that the short regular repeats are transcribed into small RNAs [72]. 

**Figure 4:**
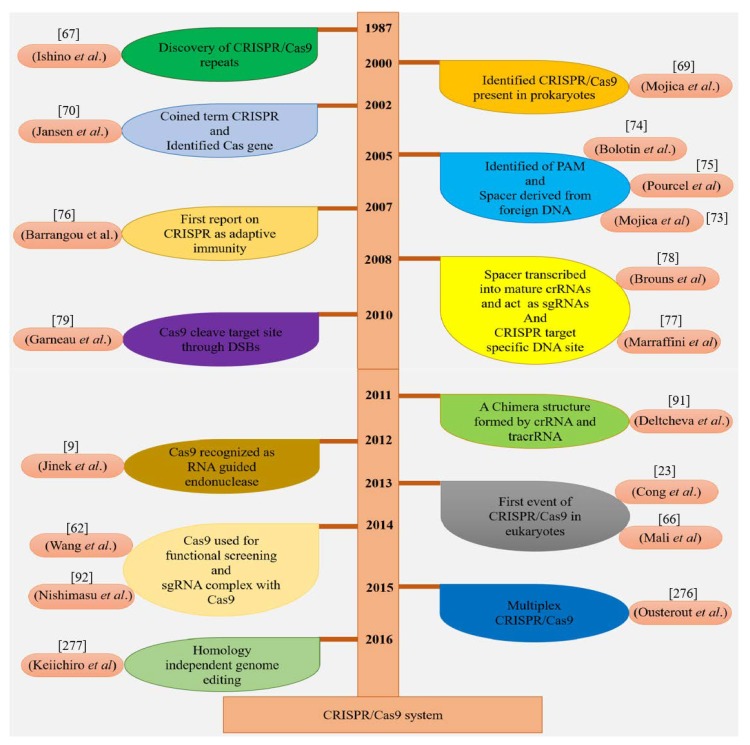
Historical chart illuminating key developments in the CRISPR/Cas9 system.

Four *Cas* genes (*Cas1*–*4*) were discovered in prokaryotes having CRISPR DNA sequences during that period [70]. From then on, many CRISPR/Cas sequences and multiple Cas proteins were identified [72]. In 2005, CRISPR spacers were discovered in plasmids and phages by three independent research groups by applying computational and sequencing technologies [73,74,75]. The function of CRISPR/Cas was still ambiguous before Barrangou et al. (2007) successfully demonstrated for the first time that CRISPR protected *Streptococcus thermophilus* from viral attack [76]. It was revealed that the CRISPR defense mechanism prevents the horizontal gene flow in *Staphylococci* [77]. In another study, it was observed that CRISPR RNAs regulate the CRISPR interference [78]. The presence of the CRISPR/Cas system in the bacterial genome was identified to cut specific sites in plasmid DNA and bacteriophages [79]. In 2011, the CRISPR/Cas machinery of *S. thermophilus* was exploited to confer immunity in *E. coli* [80]. Some of these important events are highlighted in Figure 4.

### 3.2. Architectural Organization of CRISPR/Cas9 System and Its Functions

Prokaryotic organisms such as bacteria and Archaea have a special type of defense machinery called CRISPR/Cas [81] in their adaptive immune network to protect them against the attack of viruses and phages [82].

All the natural CRSIPR/Cas networks are composed of many *Cas* genes, which are encoded by homologous palindromic repeated units, RNA-mediated endonucleases, and novel short RNAs, termed as “spacers” produced by the introduction of short mobile sequences called protospacers. The protospacers are derived from the unique spacers which move among the homologous palindromic sequences repeats when the cell is attacked by invaders. These spacers work as identifying units for the invaded cell and allow the CRISPR-Cas system to cut foreign DNA sequences. There are three steps to the CRISPR/Cas-mediated immune system, including adaptation, expression, and interference. The first step in this mechanism is adaptation, which is associated with the sequential layout of the CRISPR-array via a new spacer’s procurement. Precursor CRISPR-RNA (pre-crRNA) and *Cas* genes are expressed in the expression stage. Mature cr-RNAs are produced from precursor CRISPR-RNA using RNase III and Cas proteins in the interference event-specific targeted portion memorized by the combinative properties of both Cas proteins and cr-RNA [83]. The CRISPR motif, termed as protospacer adjacent motif (PAM), is connected with each protospacer and closely situated in the target portion of the sequence. The PAM was found to be a highly specific part of the foreign phage or virus genome but is not present on the CRISPR locus in bacterial genome [78].

The PAM sequence consisting of conserved dinucleotides is required upstream of the binding sites of crRNA for Cas proteins to recognize the target sequence [9]. The Cas proteins are unable to detect target DNA for effective cleavage during PAM site recognition. The PAM is also exceptionally crucial to distinguish between the bacteria’s own DNA and invader DNA sequences. Such features enable bacteria to defend their own DNA from nucleases [84]. In several kinds of CRISPR networks, PAM sequences are essential for Cas proteins functions, such as PAM sequence 5′-NNNNGATT which is targeted by Cas proteins in *Neissseria meningiditis* [85]. Similarly, Cas9 proteins target the PAM sequence 5′-NGGNG or 5′-NNAGAA in *S. thermophiles* [79,86] and 5′-NGG in *S. pyogenes* [9]. 

**Figure 5:**
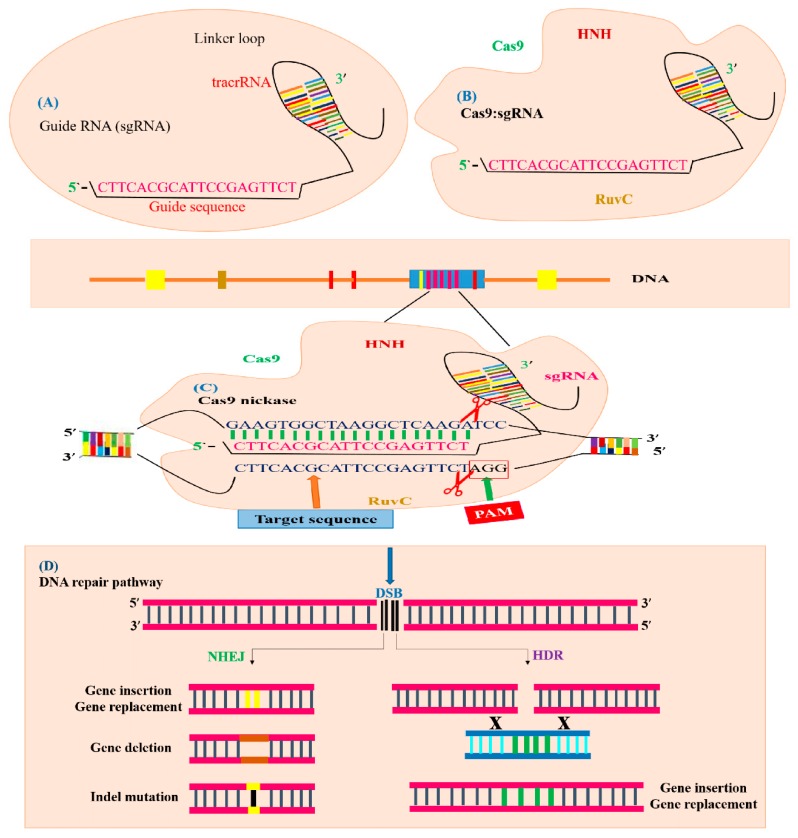
Illustration of CRISPR/Cas9-mediated GE. The CRISPR/Cas9 system is composed of sgRNA and Cas9. (**A**) sgRNA with a guide sequence (colored pink) is developed by the combination of protospacer with crRNA and tracrRNA. (**B**) Cas9 machinery combines with sgRNA to form a complex to trigger CRISPR/Cas9 editing. The Cas9 nuclease consists of two parts, depending on its function and structure. The recognition site identifies the target DNA and interacts with sgRNA. The nuclease site contains two domains RuvC-like and HNH which cleave the target DNA site non-complementary by the RuvC domain and complementary by the HNH domain to the gRNA. (**C**) The Cas9 nuclease detects the genomic target site (indicated with blue color) having a 20 bp target sequence that is homologous to seed or guide sequence (indicated with pink color), which is crucial for Cas9 activity and specificity. The specific PAM sequence (indicated with red color) is detected by Cas9: sgRNA complex and DSBs created by the Cas9 endonuclease three base pairs upstream of the PAM sequence. (**D**) Targeted mutagenesis of a desired gene is achieved by filling the DSB (indicated with black color) by means of the HDR or NHEJ mechanism. The NHEJ repair mechanism generally produces insertion (indicated with yellow color), deletion (indicated with brown color) or indels (indicated with black line) at the break point, generating targeted mutants. The HDR repair mechanism uses a template DNA sequence for homologous recombination to produce gene replacement or gene insertion (indicated with green color).

Two independent groups of scientists discovered the CRISPR/Cas9 machinery with three major kinds (type I–II–III) and two classes in host cells [87]. In 2015, Markarova and coworkers executed the comparative genomic analysis of existing data and found two further reputed types and five subtypes [88]. During the defense mechanism of CRISPR/Cas9 against invader DNA, these two classes behave differently, such as class 1 which consists of subtypes (I, III, IV) and uses many Cas proteins, while only a large Cas protein is used by the class 2 system, which has the subtypes (II, V) [89]. In the adaptation phase of the CRISPR/Cas mechanism, spacers are added by Cas1 and Cas2 proteins and pre-crRNA develop involving Cas5 or Cas6 in the type I system. The Cas6 protein is also used in the type III system for a similar process but stimulation of 3′ end is accomplished by an uncertain element. For crRNA maturation, trans-activating crRNA (tracrRNA) and RNase are utilized in the type II mechanism [90], as shown in (Figure 5A). Currently, the immune system of *S. pyogenes* operates as a type II system, which is a well-established GE technique known as CRISPR. This CRISPR/Cas9 system is modified by two major units: a non-coding chimeric RNA and Cas9 endonucleases for double-stranded (dsDNA) breaks in DNA (Figure 5B) [9]. The Cas9 protein is directed by the guide RNA (gRNA) and Cas9 proteins recognize targeted DNA in the presence of the “seed” sequence, which is produced by spacers derived from crRNA and the *S. pyogenses* Cas9 (SpCas9) 5′ NGG ′3 sequence lying closely to the target region [9]. The crRNA and tracrRNA are complementary to each other and it directs pre-crRNA to mature crRNA by means of RNase III. After the maturation of crRNA, it guides Cas9 proteins to break specific DNA sequences [91]. In 2014, Nishimasu et al. (2014) demonstrated that the SpCas9 and gRNA DNA endonuclease has unique lobes, such as an assembly composed of a target detection lobe which is attached to the heteroduplex of sgRNA: a DNA molecule and a nuclease lobe which nicked the target DNA sequence [92], as illustrated in (Figure 5C).

### 3.3. Genome Editing Mechanism of CRISPR/Cas9 System

The mode of GE is established by the healing process of the genome. After the identification of the target site, Cas9 allows sgRNA to pair with the target DNA sequence. The Cas9 endonuclease is composed of the HNH and RucV-like domain, which cuts the target DNA strands three to four bases upstream of the PAM site. The HNH domain cuts the complementary DNA strands while the RuvC domain cleaves the non-complementary to gRNA. The blunt-ended DSBs can be repaired by the HDR and NHEJ repair pathways (Figure 5D). The NHEJ is error prone and causes DNA insertion or deletion at the target sequence [23]. The expression of sgRNA as pair, NHEJ mechanism came up with large deletions. The large deletions in chromosomes were attained by the NHEJ mechanism utilizing co-expressed sgRNAs. The HDR repair mechanism is only operational when a specific homologous target site is available with respect to the DSB site. In plants, through GE, many outstanding repairs were achieved via HDR, such as gene replacement, DNA correction, and targeted knock-in [93,94].

Biolistic and *Agrobacterium*-mediated transformation can be applied to transfer the sgRNA and Cas9 protein into desired cells [95]. GE by CRISPR/Cas9 is heavily dependent on the choice of sgRNA promoters and ubiquitous expression of the Cas9 enzyme. Universal *CaMV35S* RNA polymerase II promoters have been extensively used for Cas9 expression in plants. Similarly, for sgRNA expression, U3 or U6 RNA pol III promoters are applied [96]. The expression level of sgRNAs is significantly greater in endogenous promoters as compared to exogenous promoters [97]. Moreover, sgRNA expression is guided by U6 promoters which were derived from monocotyledonous or dicotyledonous varieties and can only be used in monocot or dicot plants [98]. For successful integration of CRISPR/Cas9 machinery in plant nuclei, Cas9 proteins must join with nuclear localization signals [96].

**Figure 6:**
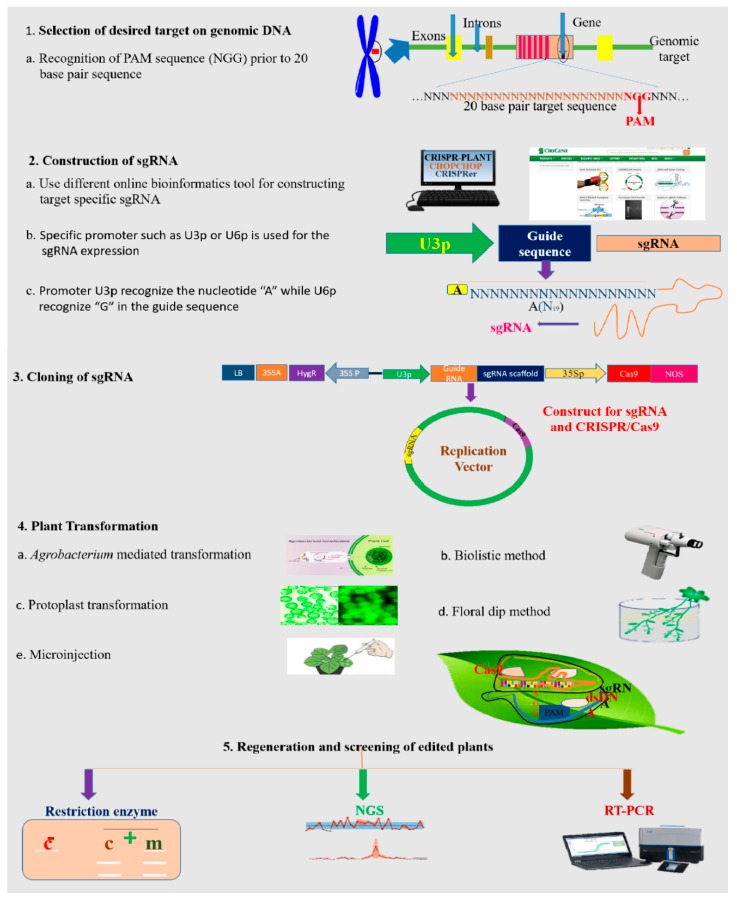
Basic steps in the workflow of CRISPR/Cas9-based genome editing.

To bind the target DNA by synthetically developed short gRNA sequences of approximately 20 nucleotides, the mechanism of CRISPR/Cas editing demands the PAM 5′ NGG motif for the Cas9 enzyme to cleave 3–4 bases in the target DNA sequence after the generation of the protospacer [9]. There are two domains of Cas nucleases which have the ability to cut one strand of DNA like the HNH domain and RuvC-like domain. Simple steps involving the execution of the CRISPR mechanism are recognition of the PAM sequence; sgRNA development; cloning of sgRNA; transformation into the host cell; selection of transformed individual organisms; and edited lines confirmation, as described in Figure 6.

## 4. CRISPR/Cas9-Mediated GE in Plants

To date, numerous efforts have been successfully employed for targeted gene editing in model as well as in major crop plants via the CRISPR/Cas9-based GE toolbox. Many factors have been reported that affect the editing ability of the CRISPR/Cas9 system including targeted DNA, GC contents, Cas9 codons, sgRNA structure, and expression of cas9 and sgRNA. All these factors must be highly optimized to achieve greater efficiency of CRISPR/Cas9 system [24]. 

### 4.1. Designing the CRISPR/Cas9 Delivery System

In the past, numerous attempts have been made at gene editing in plants, but the efficiency of CRIPSR/Cas9 was low [25,26]. With the improvements in technology, many highly efficient vector delivery systems for CRISPR/Cas9 have been designed for plant GE, such as supersession of viral infection, gene disruption of cis-elements, genomic deletion, gene knockout, and multiplex genome editing. Continuous progress in this editing toolkit has allowed more precise, accurate, and targeted delivery of the Cas9 system into plant cells, which include discovery of new Cas9 variants, efficient screening methods for knockout mutants, vector selection, and construction and employment of the most appropriate delivery system for the Cas9 expression cassette. In this section, we describe the construction, screening, and delivery of the CRISPR/Cas9 system into plant cells.

### 4.2. Cargo-Vectors for the CRISPR/Cas9 System 

Two kinds of vector systems are used in CRISPR/Cas9-mediated GE, such as a single-vector system and a binary-vector system. A binary-vector system has been utilized for many years because of its ability for fast primary testing. Any specific vector having several gRNAs and a Cas9 protein expression cassette already constructed in it can be applied for plant transformation. Different structural construct of gRNAs can be utilized for numerous Cas9 proteins to design a unique gRNA: Cas9 nuclease, which allows more accuracy and easiness in experimental design. A single vector harboring both expression cassettes of gRNA and Cas9 protein is becoming more promising. Generally, in a single-vector system, RNA polymerase III-driven promoters (U6/U3) are designed for gRNA expression, whereas ubiquitin and *CaMV35S* promoters based on RNA polymerase II are exploited for *Cas9* gene expression. Continuous advancements in CRISPR/Cas9 permit researchers to develop smarter vector systems to regulate expression of gRNA and the *Cas9* gene. Recently, some new adjustments were made in the single-vector system, such as single polymerase II and dual polymerase II promoters. Single polymerase II vectors are applied to govern the expression of gRNA and the *Cas9* gene at the same time, while the dual polymerase II vectors exploit two different promoters to drive the expression of gRNA and the *Cas9* gene. The addition of all these latest technologies in the CRISPR/Cas9 delivery system assists to decrease the vector length, which eventually, improves the transformation efficiencies [99].

### 4.3. Bioinformatics Tools for Designing the CRISPR/Cas9 Construct

One of the most crucial steps for highly precise GE is to design an sgRNA construct for CRISPR/Cas9. To date, numerous bioinformatics tools have been developed and are available online for sgRNA designing. There are many accessible online tools with plant databases and which permit the design of sgRNA for identification of new target sites [100], as shown in Table 2. For example CRISPR Design (http://www.genome-engineering.org) was developed by Zhang and colleagues for designing sgRNA and it also assists in assessing off-target mutation [101]. In 2014, Xie and coworkers successfully developed a web tool named CRISPR-PLANT (http://www.genome.arizona.edu/CRISPR) in order to design efficient sgRNA constructs for CRISPR/Cas9-based GE [102]. For example, a novel web tool was developed by Michano and colleagues for rapid detection of target loci in soybean for CRISPR/Cas9-mediated GE [103]. Similarly, CRISPR-P (http://cbi.hzau.edu.cn/crispr/) eases the designing of sgRNA for every plant having an available sequenced genome and also helps to evaluate off-targets [104]. 

### 4.4. Construction of the sgRNA Expression Cassette

Construction of a unique sgRNA expression cassette is the most important step in CRISPR/Cas9-mediated gene editing, and it works as a guide system for the Cas9/sgRNA complex consisting of 98 nucleotides with a 20 nucleotide target sequence [92]. In plants, RNA polymerase III is used to transcribe sgRNA and its expression is mostly governed by U3 or U6 promoters [93]. As the expression cassettes of sgRNA:U3/U6 promoters are very small in length, approximately ~300–600 bp, overlapping PCR or adaptor ligation can be applied to construct these expression cassettes [123]. In 2015, Ma and colleagues developed a robust cloning-free approach for sgRNA expression cassette development based on the PCR technique. Gibson assembly or the Golden Gate cloning strategy was used for direct cloning of sgRNA expression cassette into binary vectors for the CRISPR/Cas9 system [24]. In another approach, Gao and Zhao utilized a ribozyme mechanism to generate sgRNA by transcription of pre-RNA through RNA polymerase II, whereby inducible or constitutive promoters can be ligated to obtain the desired function of sgRNA [124]. 

### 4.5. Construction of Cas9 Expression Cassettes

Cas9 is composed of 4107 bp of coding sequence. For Cas9 nuclear localization in eukaryotes, the Cas9 coding sequence must be fused with the nuclear localization signal. Plant usage–bias codons have been used to design highly efficient and optimized Cas9 expression cassettes for improved GE in plants [125]. For example, utilization of codon-optimized Cas9p in rice and Gramineae family has been improved by enhancing GC contents [24], which imitate the genes from the Gramineae family [126]. Commonly, constitutive promoters like 35S *Cauliflower mosaic virus* (CaMV) and ubiquitin from *A. thaliana*, rice, and maize can govern the expression of Cas9 in dicots and monocots for highly targeted gene editing using callus-based transformation approaches. 

### 4.6. Transformation Approaches for CRISPR/Cas9-Based Vector Delivery into Plants

For CRISPR/Cas9-mediated GE, cargo–vector harboring the expression cassettes of both sgRNA and the *Cas9* gene must be carried to targeted sites in plant cells. For cargo–vector transformation, floral dip and biolistic approaches are generally executed. Nowadays, advanced strategies like ribonucleo-protein complex, plasmid delivery, and virus-mediated delivery systems are applied for plant transformation. There are certain limitations in using the virus-mediated delivery system, but several studies have been carried out in plants using the virus delivery system [127,128]. A transient expression system is commonly used by researchers to transfer the vector into protoplast for analyzing the efficiency and feasibility of the CRISPR/Cas9 toolkit [129]. Biolistic and PEG-mediated transformation techniques can be used for direct delivery of *Cas9* gene expression cassettes [130]. However, it is difficult to regenerate plants from protoplast due to the heritable targeted mutations, which poses a major drawback associated with this approach in many plant species. *Agrobacterium*-mediated transformation is a highly efficient approach for stable transformation of the CRISPR/Cas9 system in dicot and monocots [131,132].

### 4.7. Strategies for Mutant Screening

The CRISPR/Cas9 system is a groundbreaking innovation in GE technology for developing desired mutants and numerous mutant libraries have been created by the CRISPR/Cas9 system so far, such as the genomic-scale mutant library for tomato [133] and rice [134,135]. As the applications for GE approaches are increasing day by day, scientists are required to screen huge numbers of mutants, using a strategy that includes the detection of off-target and on-target edits and which later removes the transgenes in edited plant off-springs.

To overcome the limitations associated with mutant screening, different techniques have been developed, including annealing at critical temperature polymerase chain reaction (ACT-PCR) [136], high-resolution melting analysis (HRMA) [137], polyacrylamide gel electrophoresis (PAGE)-mediated genotyping [138], T7 endonuclease I (T7EI) approach [139], and restriction enzyme site loss technique [140]. There are certain pros and cons for each technique and they are centered on genotyping differences. A mutant can be detected rapidly when it has a clear evident phenotype. For example, a visible albino phenotype was observed when a gene phytoen desaturase mutated via the CRISPR/Cas9 system. It was applied as a phenotypic marker to detect rice- and tobacco-edited plants [141,142]. Additionally, transgenic plants can also be screened using some herbicide/antibiotic selectable markers [141,143]. But making a connection among visual phenotypes and targeted genes is the only challenge associated with phenotyping-mediated screening [135]. In other approaches, high-throughput sequencing is highly efficient and precise strategy to screen all the mutants generated by the CRISPR/Cas9 system [144]. For the detection of DNA-free plants edited by CRISPR/Cas9, whole genome sequencing is quite beneficial and helpful [145]. 

## 5. Recent Breakthroughs in CRISPR/Cas9-Mediated Genome Editing in Plants

The GE tool of the CRISPR/Cas9 system has gained remarkable importance in agriculture; however, there are some drawbacks which limit its application in plant GE. The major concerns related to this technology are non-specific off-targets; HR inefficiency; PAM sequences constriction; cargo-vector inefficiency; and many others. With the advancement of plant biotechnology many unique innovative steps are regularly incorporated into the GE network to tackle these challenges. 

### 5.1. CRISPR/Cas DNA as Cargo-Delivery Vector

The conventional cargo-vectors for GE have certain limitations which hinder the accurate editing mechanism in plants. The CRISPR/Cas DNA-based cargo-vector is now extensively used to overcome the drawbacks in plant GE.

#### 5.1.1. Stable Expression

The abovementioned approaches of vector delivery into plant cells, such as biolistic and *Agrobacterium*-mediated transformation, are used to deliver the CRISPR/Cas DNA, and by screening the mutants, the DNA is inserted into the target location in the plant genome and carry out the editing phenomena. Currently, there are numerous applications of this system in plant GE. In 2016, Gao and colleagues incorporated a fluorescent gene in the expression cassette of CRISPR/Cas9 [146]. Recently, an interesting strategy was developed in which *BARNASE* and *CMS* were used as suicide genes to kill embryos and pollens containing the transgene of T0 plant progeny [147]. 

#### 5.1.2. Transient Expression 

Another delivery strategy that can be employed to obtain transgene-free edited plant is the CRISPR transient expression system. In this technique, the screening of mutants using selectable markers (antibiotic/herbicide) is omitted to regenerate the edited progeny without the incorporation of foreign genes into the plant genome. In 2016, Zhang and co-workers successfully executed this strategy for the first time in wheat to obtain transgene-free plants [148]. Furthermore, DNA base editors such as adenine and cytidine base editors have also been transferred by the transient expression approach for attaining DNA-free base substitutions [149,150]. *Agrobacterium*-mediated transformation-based transient expression has also been successfully developed in tobacco [142], and protoplast transformation-based transgenes-free expression can also be achieved in tobacco and potato protoplasts [151,152].

### 5.2. DNA-Free Genome Editing Through Ribonucleoproteins (RNPs)

Foreign DNA-free GE is another approach in the CRSIPR/Cas9 toolkit to produced transgene-free plants [153]. In conventional approaches to genetic engineering, the foreign DNA with editing components is incorporated into the host organism. Due to the random incorporation, the genetic modifications can be unpredictable. Even if the expression cassettes are abolished, the fragments of foreign DNA can still be integrated into the host genome and cause mutations [154]. Furthermore, the introduction of genetically modified organisms has increased globally [155]. So, there is an increased demand to produce transgene-free plants. To develop DNA-free genome-edited plants, particle bombardment and protoplast transformation techniques have been employed. 

Transgene integration may be reduced by using the CRISPR/Cas DNA transient expression system, but it does not entirely eliminate it; additionally, the discarded DNA portion may still get incorporated into different non-targeted locations within the plant genome. To escape the shortcomings of mRNA and plasmid-based expression system of sgRNA/Cas9, a most competent transgene-free editing strategy was established by designing the RNPs sgRNA/Cas9 system in plants [156,157,158]. Hence, sgRNA/Cas9 RNPs have a greater ability to produce DNA-free edited plants with low off-target frequency and is more efficient than a plasmid-mediated editing system. The RNP-based system does not need transcriptional and translational apparatuses for creating nicks in the target sites and, after cleaving, it is then disintegrates itself. In 2015, Woo and colleagues performed DNA-free GE for the first time in rice, tobacco, lettuce, and *Arabidopsis* using the RNPs system [156]. Potato, apple, and grape explants were subjected to targeted mutations carrying CRISPR/Cas9-mediated RNPs [159,160,161]. In addition, DNA-free GE in maize and wheat has been developed by particle bombardment-mediated transformation of RNPs and Cas9 proteins into cells [58,148,162]. Recently, a new CRISPR/Cas variant Cpf1 has been added to the RNP-based GE toolkit, carrying AsCpf1/crRNA and LbCpf1/crRNA RNPs into tobacco and soybean [163]. However, in several cereal crops protoplast regeneration is a bigger task, hence, the biolistic-mediated RNP editing system is the most appropriate technique for GE in plants. The delivery of RNP-mediated CRISPR/Cas9 machinery has been demonstrated by two different groups in wheat and maize [145,146]. The discovery of a DNA-free editing system will surely simplify the GE of plants and helps to commercialize the edited plants in the future.

### 5.3. CRISPR/Cas9 Toolbox: Ways Toward Precise Editing 

#### 5.3.1. Base Editing

As compared to DSB-governed GE, single-nucleotide modification at a specific site of the genome is called base editing, and is not based on donor DNA or an HDR mechanism and also does not require DSB generation, which provides a simple, highly accurate, and universal mechanism for editing a single base at a target site. Thus, base editing with the CRISPR/Cas9 tool is gaining interest for precise targeted gene editing in plants [164]. Currently, the use of the HDR repair mechanism with donor DNA for DSBs has been found to be less effective in contrast to NHEJ repair with a template-free system, posing a great hurdle in plants for base substitution. Genome-wide association studies (GWAS) have demonstrated that crop plants having a single nucleotide insertion/deletion are more significant for screening the elite germplasm [16]. Therefore, powerful tools are required immediately for generating accurate base editing in crop plants [165]. The CRISPR/Cas9 is an exceptional technique for precise substitution of a single base in target DNA [166]. The CRISPR/Cas9-directed base editing strategy has used the gRNA system, which is homologous to the natural CRISPR system. But in case of a cytosine base-editor (CBE) system, modified Cas9 endonucleases called nickase (nCas9) are used as compared to the natural CRISPR system. These nCas9 proteins, in addition to dead Cas9 proteins fused with an enzyme having base cleaving activity such as cytidine, are converted to uridine by the cytidine deaminase [149,167]. Recently, an effective base-editor 3 (BE3) platform was developed, which involves the merger of APOBECI known as rat cytidine deaminase and which has been extensively employed for GE in many organisms including plants [168]. In addition, several improvements in the BE3 system has allowed modifications in PAM sites to enhance its editing specificity and accuracy [168]. Likewise, three cytidine deaminase orthologs such as human APOBEC3A [150,169], human AID [170], and lamprey PmCDA1 [167] have been fused with nCas9 to attain highly precise C-to-T substitution. For example, a plant CBE system based on APOBEC3A has been widely applied for C-to-T substitution in potato, rice, and wheat [150,171]. In rice and *Arabidopsis*, CBE has been used to create point mutations. In addition, CBE can also be applied to generate non-specific mutations that manipulate the desired gene and disrupt its function. CBE was found to be precise and more accurate than SSN-mediated editors, producing rare if any indels [172].

Similarly, adenine converts to inosine by adenine deaminase [168]. In wheat and watermelon, this strategy has been adopted to develop herbicide-resistance plants [50,173]. Yan and colleagues identified a fluorescence-tracking mechanism in rice which converts the adenine to guanine by a single-base editing system [174]. An adenine base editor (ABE) was developed for multiplex base substitutions in rice [175]. Similarly, ABE was applied to study the germline transmission and preferred phenotypic changes in *Arabidopsis* [176]. Recently, Li et al. (2018) upgraded the ABE for generating base editing in wheat and rice plants. They have also successfully developed an herbicide-resistant rice by producing the point mutation [177]. So, GE has been provided novel dimensions by base-editing tools, widening its prospective applications by manipulating desired nucleotides in the plant genome. 

#### 5.3.2. Multiplex Genome Editing

In plants, cellular processes are fine-tuned by several redundant genes. Sometimes, mutating a single gene may not confer a desired phenotype because of the compensation effect produced by other genes in same gene family. Hence, an upgraded editing system with improved efficiency is needed for multiplex gene editing in plants. In CRISPR/Cas9-mediated multiplex GE, many sgRNA cassettes can be designed by using single or multiple promoters into a single-vector system [108,178]. In 2013, Mao and coworkers designed two sgRNAs for two homologous of *magnesium-chelatase subunit I* (*CHLI*) having function in the photosynthesis mechanism, and it successfully transformed the vector in *Arabidopsis thaliana.* The result showed the albino phenotype in plants in which both genes were disrupted [179]. In another study, four subunits of katanin p80 were mutated in *A. thaliana* using multiplex genome editing. For this, three sgRNA expression cassettes were designed for simultaneous gene editing and the results demonstrated the dwarf phenotype in quadruple-mutant plants [180].

The group of Xie reported the editing of eight genes simultaneously by designing multiple sgRNA expression cassettes. An endogenous t-RNA-processing platform was used for the expression of multiple sgRNAs. All the sgRNAs were released after the nick produced by endogenous t-RNA-processing-based RNase [181]. Similarly, this t-RNA-based strategy has also been efficiently demonstrated in *Zea mays* [182]. A multiplexing system was developed by Tang and colleagues in which hammerhead self-cleaving ribozyme was applied. Additionally, the same promoter Po1II was used for the expression of multiple sgRNAs that govern Cas9 activity. Ribozyme cleavages separated sgRNAs and Cas9 after transcription and released functional sgRNAs and Cas9 [183]. Furthermore, the ability of the CRISPR-Cpf1 system was harnessed for multiplex GE in rice. A single promoter was applied to produce a construct composed of numerous repeated units of crRNA attached with a target sequence. A target repeat sequence was recognized by Cpf1 and produced cleavage, which resulted in releasing of crRNAs [184]. Hence, CRISPR/Cas9-mediated multiplex GE is a convenient approach for knocking out multiple genes at once and helping to decipher the function of a desired gene family that regulates multiple biological networks. Moreover, it is also beneficial in finding out the epistatic association among genes in numerous genetic processes. 

### 5.4. Beyond Cas9: New Cas Variants Broadening the CRISPR Toolbox

The CRISPR/Cas9 system which originated from *Streptococcus pyogenes* has some drawbacks which hinder its editing activity like multiple incompatible off-targets due to the gRNA mismatches. Thus, several changes have been made to enhance the editing efficiency and to minimize the off-target nicking of Cas9 enzymes including SpCas9n (Cas9n) [23], Dead cas9 (dcas9) [185], and *FokI* Cas9 (fCas9) [186,187]. Various bacterial species have been used for the extraction of Cas9 proteins having novel- and stretched-PAM sequences that can help in enhancing the non-target cleavages. *Neisseria meningitides* have unique a CRISPR/Cas machinery named Nmecas9 which is specific for 8-mer (5′-NNNNGATT) PAM sequence targets that can minimize the chance of off-target cleavage and enhance specificity [188]. Besides, of the other identified orthologs of Cas9, SpCas9 has been most commonly used for GE. SpCas9, derived from *Staphylcoccus aureus*, detects the 6-mer PAM sequence (5′-NNGRRT) [189]. The modification of SpCas9 has been carried out which targets the PAM sequence (5′ NGA) and edits the target gene efficiently [190]. Besides SpCas9, the shorter length of SaCas9 permits it to overcome the delivery challenges faced by SpCas9 in utilizing the multi-dimensional adeno-virus cargo vectors [189]. The CRISPR/SaCas9 system has been efficiently used to edit many plant genomes such as citrus, rice, tobacco, and *A. thaliana* [191]. Furthermore, *Streptococcus thermophilus*-derived St1Cas9 and St3Cas9 have also been employed for CRISPR-mediated GE [191]. Different types of tracrRNA and crRNA are used by these orthologs to identify different PAM sites [192].

#### CRISPR/Cpf1 System

Recently, *Francisella novicida* was studied to discover the Class II type CRISPR-Cpfl system [193], recently named Cas13 [194]. In comparison to Cas9 for cleavage and production of cohesive ends, Cpfl needs a single RNA-guided complex having 4–5 nucleotides 5′-overhangs. The CRISPR-Cpfl system has been used successfully with none or fewer off-targets in both animals and plants. In 2016, the CRISPR/Cpf1 tool was effectively applied for GE in plants [195]. Due to the exceptional properties of Cpf1, type V CRISPR/Cpf1 has been considered as another powerful technique for plant GE [196]. The CRISPR/Cpf1 machinery like the conventional CRISPR/Cas9 system is formed by the two major elements: one Cpfl nuclease for target specificity and the other one for target sequence identification called crRNA. Although, in contrast to the Cas9 network, which recognizes PAM sequences with G-rich contents (5′-NGG-3′), the Cpf1 recognizes a PAM sequence (5′-TTN-3′) having T-rich contents [194]. Furthermore, CrRNA and tracrRNA interaction is not needed in the Cpfl system, although it is necessary for the Cas9 technique. A size of about 42 to 44 crRNA is required in the CpfI system, which is smaller than that of gRNA [194]. In rice and tobacco, targeted mutagenesis has been carried out through the CRISPR/Cpf1 mechanism derived from *Francisella novicida* (FnCpf1) [195]. In rice, *Lachnospiraceae*-derived Cpf1 (LbCpf1)-mediated targeted mutations have been reported [197,198]. Similarly, LbCpf1 and FnCpf1 nucleases have great potential for precise GE for specific gene addition via HR mechanism [199].

## 6. Applications of CRISPR/Cas9 in Plant Breeding 

Climate change and rapid increases in the world’s population are two major concerns that threaten agriculture production and food security globally [200]. Several biotic stressors (bacteria, viruses, fungi, insects, nematodes, etc.) and abiotic stresses (drought, salinity, heat, cold, waterlogging, etc.) hamper crop production and compromise food security around the world. Crop breeders are striving hard to develop climate-resilient, stress-tolerant crops with better quality and increased production [201]. Thus, the CRISPR/Cas9 system has numerous applications for the functional genomic research of plant genes that play a crucial role in genetic improvement of many significant agronomic traits. Especially, the knockout of some genes can encourage superior traits including disease resistance, adaptation to various abiotic stressors, nutrient usage, and yield improvements. Thus, CRISPR/Cas9-mediated GE has great potential in plant breeding for crop improvement.

### 6.1. CRISPR/Cas9 System for Plant Disease Resistance

Virus, bacteria, fungi, nematodes, and insects are the major causal agents inducing biotic stressors and crop yield reduction. Moreover, the persistent upsurge in several new strains of lethal pests make the battle very challenging against these pathogens [202]. Thus, to protect agriculture from the devastating impact of biotic stressors, it is very crucial to understand the plant–pathogen interaction [203]. GE strategies have been successfully applied to explore plant–pathogen interactions and mechanisms underlying plant responses against pathogen attack.

CRISPR/Cas9-mediated GE can be employed directly to disrupt disease-causing genes, known as “S-genes” and develop disease-resistant crops. For example, targeted knockout plants for the ethylene-responsive gene *OsERF922* were generated via the CRISPR/Cas9 tool, which showed reduced blast lesions and increased resistance against rice blast caused by *Magnaporthe oryzae* [204]. Likewise, bacterial blight-resistant plants were produced by targeted mutagenesis of the *SWEET13* gene [205]. CRISPR/Cas9-based mutagenesis was applied to the promoter region, and transcription factor (TF) of canker *CsLOB1* in *Citrus paradise* was identified. Due to the presence of such mutations, two mutant lines *D_LOB_10* and *D_LOB_9* with high mutation rates have been produced. The frame-shift mutation and disruption of the *CsLOB1* gene improved resistance against *Xanthomonas citri* [206]. Peng and colleagues reported the editing of effector binding elements (EBEs) by the CRISPR/Cas9 system in the *CsLOB1* gene promoter region to increase disease resistance in *Citrus sinensis* against *Xanthomonas citri* [207]. 

In wheat protoplasts, the CRISPR/Cas9 technique was applied by Shan et al. to edit the *TaMLO* gene [141] and produce wheat lines resistant to powdery mildew caused by *Blumeria graminis* f. sp. *Tritici* [62]. In another study, CRISPR/Cas9-mediated multiplex GE was performed to mutate three homologs of the *EDR1* gene to develop resistance against powdery mildew in wheat [208]. Similarly, CRISPR/Cas9-based mutants of *MLO* were produced in tomatoes which conferred resistance against powdery mildew [209]. 

It has been estimated that about half of plant diseases are caused by virulent viruses, which result in heavy crop losses globally [201]. Gene-targeting efficiencies were improved many folds by DNA virus amplicons. Geminiviral-based DNA replicons of wheat was utilized for transient expression of the CRISPR/Cas9 system against *wheat dwarf virus* (*WDV*), in hexaploid wheat and 12 fold upregulation was observed in ubiquitin gene expression [210]. Stable over-expression of sgRNAs and Cas9 that particularly target the genome of the Gemini-virus to prevent its growth has been applied for virus-resistant crop breeding programs [211,212,213]. Furthermore, the CRISPR/Cas9 system can also be used to mutate viral genomes in addition to tackling diseases caused by them [201]. The efficiency of CRISPR/Cas9-mediated viral GE can be increased by using virus promoters to govern sgRNA/Cas9 expression cassettes [211]. Recently, a new ortholog of Cas9 has been discovered in *Francisella novicida* (FnCas9) to edit RNA virus genomes. FnCas9 has successfully inhibited the replication of the tobacco mosaic virus as well as the cucumber mosaic virus and provides immunity against them [214]. Therefore, CRISPR/Cas9-mediated GE is an exceptional tool to improve genetic make-up and enables them to combat various pathogens. A list of recent studies indicating the significant success of the CRISPR/Cas9 system against various plant diseases is compiled in Table 3.

### 6.2. CRISPR/Cas9 for the Production of Climate Smart Crops

The CRISPR/Cas9 technology has been extensively applied in major crop plants such as wheat, rice, maize, cotton, soybean, tomato, and potato to cope with various abiotic stressors. Development of climate smart abiotic stress-tolerant crops via the CRISPR/Cas9 tool has modernized plant breeding programs. Major events for crop improvement via CRISPR/Cas9 are described in Table 4.

For example, in wheat protoplast, two genes related to abiotic stress, *TaDREB3* and *TaDREB2*, have been studied using the CRISPR/Cas9 technique. With a T7 endonuclease assay, the expression of mutated genes has been confirmed in approximately 70% of transfected protoplasts. The mutated plants showed increased tolerance against drought as compared to wild cultivars [221]. Three rice genes named mitogen-activated protein kinase (*OsMPK2*), phytoene desaturase (*OsPDS*), and betaine aldehyde dehydrogenase (*OsBADH2*) have been edited using the CRISPR/Cas9 technique. For transformation of CRISPR/Cas9 machinery, particle bombardment and protoplast transformation methods were used and revealed that these genes are responsible for regulating many abiotic stressors [26]. 

To protect plants from abiotic stressors, plant annexins play a major role. The annexin *OsAnn3* gene in rice has been studied under cold stress and its function was determined in edited knockouts developed by the CRISPR/Cas9 system [222]. Similarly, the gene *SAPK2* was mutated to study the stress tolerance mechanism in rice. The results revealed that the expression level of *SAPK2* was enhanced under drought and salinity stress conditions [223]. Drought tolerance in transgenic maize was enhanced by the overexpression of *AGROS* genes and they are of great prominence for maize breeding. To identify new allelic variants, CRISPR/Cas9 was applied to mutate the *ARGOS8* gene [224]. Curtin and colleagues carried out CRISPR/Cas9-based knockout mutagenesis of two genes *Drb2a* and *Drb2b* and found that these genes regulate salt and drought tolerance in soybean [225]. In tomato, important signaling molecules, i.e., mitogen-activated protein kinases (*MAPKs*) that respond against drought stress by protecting the membrane of cells from oxidative destruction and regulating genes transcription to tackle drought stress. The association of the *SlMAPK3* gene in controlling the drought tolerance mechanism has been reported in tomato by creating knockout mutants of the *SlMAPK3* gene under drought stress through the CRISPR/Cas9 system [226].

Many important traits like stress tolerance and crop yield are controlled by multiple genes. Many studies have been carried out to locate the quantitative trait loci (QTLs) that are controlling important traits in crop improvement programs. For the development of better performing varieties, such QTLs have been transferred into the elite lines. But this introgression is laborious for closely associated QTLs and if non-target regions are introduced into best performing varieties, it may produce many deleterious effects. Conversely, CRISPR/Cas technology can be a fascinating approach to generate and examine targeted mutagenesis. Using a CRISPR/Cas9-mediated QTL editing approach, the functions of grain number QTLs (*Gn1a*) and grain size (*GS3*) in rice varieties were examined [227]. Hence, the above studies revealed that CRISPR/Cas9-based GE has massive potential for the development of climate-resilient crops. 

### 6.3. Crop Yield and Quality Improvements via CRISPR/Cas9

Two other important agricultural traits are crop yield and quality that need to be improved through the CRISPR/Cas9 system to ensure food security worldwide (Table 5). Crop yield is a complex, multi-genic, and quantitative trait that is influenced by several features. The CRISPR/Cas9 technology has demonstrated its worth for quick yield improvement in crops.

In many studies, the CRISPR/Cas9 technique has been used to knockout the genes that negatively regulate yield related-traits including tiller number (*OsAAP3*), panicle size (*OsDEP1*, *TaDEP1*), grain weight (*TaGW2*, *TaGASR7*), grain size (*OsGS3, OsGRF4*), and grain number (*OsGn1a*). The results demonstrated that CRISPR/Cas9 is an efficient technology for improving crop yield [236,237,238,239,240]. A multiplexing GE strategy has been employed to mutate three genes simultaneously including *GS3*, *GW2*, and *GW5*, and *TGW6* which headed towards trait pyramiding and enhancing grain size and weight in rice [241]. Similarly, Li and coworkers applied the CRISPR/Cas9 system to knockout three yield-related genes, *Hd2*, *Hd 4* and *Hd5*, which resulted in early heading in rice [242]. It was reported that the *OsSWEET11* gene has a crucial role in grain filling and sucrose transportation. So, the CRISPR/Cas9 system was applied to disrupt the *OsSWEET11* gene, which led to decreased sucrose concentration and reduced grain weight. This study suggested that the overexpression of this gene may be beneficial for maximizing rice yield [243]. In wheat, CRISPR/Cas9-mediated GE knockout of the *GASR7* gene increased kernel weight [148]. Recently, a new approach has been established for gene identification on a large-scale that assists in examining the complex quantitative traits, including yield, by integrating the CRISPR/Cas9 tool, whole genome sequencing, and pedigree analysis. In a study, 30 varieties of “Green Revolution miracle rice” were subjected to genome sequencing and 57 genes controlling yield-related traits were screened. Knockout mutants of those 57 genes were created using the CRISPR/Cas9 technique. Phenotyping indicated that several genes are crucial for regulating yield-related traits in rice [244]. 

There are also many applications of CRISPR/Cas9 technology for quality improvement in crops such as storage quality, nutritional value, fragrance, and starch contents. For example, the cooking and eating quality of rice has been improved by mutating the *Waxy* gene using CRISPR/Cas9 [245]. The nutritional value of rice has also been improved by knocking out the *SBEIIb* gene which resulted in more amylose synthesis [246]. Similarly, the starch synthase gene *GBSS* was mutated via CRISPR/Cas9 in potato. The mutated lines showed decrease levels of amylose and enhanced the concentration of the amylose/amylopectin ratio [152]. In 2018, Sanchez and colleagues, carried out CRISPR/Cas9-mediated GE of the gluten-encoding gene family *α–gliadin* to produce low-gluten wheat [247]. To improve oil composition of soybean, the CRISPR/Cpf1 system was employed to disrupt the *FAD2-1B* and *FAD2-1A.* The results revealed high-yielding soybean plants with improved levels of oleic acid [163]. Moreover, it was reported that the zein protein has been reduced by 12.5% in kernels by disrupting the *PPR* and *RPL* genes in maize. The mutated plants indicate increased production levels of healthy tryptophan and lysine in maize [182]. Sorghum nourishment quality has been improved by targeting *k1C* genes which were responsible for poor digestibility and hindered production of important amino acids [248]. Recently, some other studies for quality improvement have been carried out via the CRISPR/Cas9 system, such as *Brassica napus* with high oleic acid concentration [249], long shelf life of tomatoes [250], and increased lycopene levels in tomato [251].

In summary, the above described studies reveal that CRISPR/Cas9-mediated modern breeding techniques can be utilized to attain valuable mutations for improving crop yield and quality.

## 7. Regulatory Affairs of Genome-Edited Crops

Since the development of the first genetically modified organism (GMO) in 1995, firm rules and sanctions have been imposed to regulate GM crops worldwide. In most European countries, GM crops are still banned for commercial production and release of GM crops in the field and consumer market is prohibited. Similarly, GE non-transgenic crops may also be banned if regulatory bodies consider them as GMOs. GM crops continue to provoke extensive public misunderstanding and mistrust despite 22 years of commercialization and cultivation on 189.8 million hectares in 2017 with approximately US$18.2 billion economic gains in 2016. Globally, 82% of the total crop area for soybeans, 68% for cotton, 30% for maize, and 25% for oilseed rape were planted with GM varieties in 2014. Despite high adoption rates by farmers, the cumbersome regulatory processes and delayed cultivation approval procedures have reduced the value of innovation “the GM crops”. 

Quick action may be needed for strict legislation to distinguish between GE and transgenic crop. Modern breeding technologies, particularly GE, are highly feasible alternative options to GM crops and involve a reduced degree of regulatory oversight. New discoveries in GE technology and continuous progress in delivery systems that do not need to insert any specific foreign DNA in host cells for crop improvement may strongly challenge the legislative laws regulating the transgenic crops [130]. 

Traditional plant breeding approaches mainly depend on chromosomal modification via homologous recombination. Selection and crossbreeding have been applied for many years to screen the best performing varieties. Conventional plant breeding techniques have been used for several decades to detect novel traits and introduce them into individual plants for desired results. Traditional breeding has successfully developed many new cultivars, but these approaches need rigorous and continuous selection for many generations [256]. Genetic variability has significantly decreased due to the progressive evolution of many major crops through traditional breeding [257]. Therefore, modern plant breeding approaches have become essential to overcome the certain limitations of traditional breeding such as self-incompatibility, long generation time, heterozygosity, polyploidy, and time consuming.

Mutation breeding and engineering of transgenic plants are other crucial strategies used for crop improvement [258]. Conventional mutagenesis has helped to produced genetic variations that ultimately help in improving food quality and crop yield. For genetic analysis, natural or artificial mutagenesis has been induced using chemical and physical mutagens like ethyl methanesulfonate (EMS) and gamma rays [259]. Screening of large numbers of mutants is the biggest challenge. Such laborious, time-consuming, and untargeted breeding platforms cannot maintain pace with global food demands [258]. Foreign genes have been transferred into the elite crop lines to obtain desired traits through transgenic breeding. As compared to traditional breeding, transgenic techniques eliminate all crossing barriers and have increased genetic variability. Transgenic technology has provided enormous opportunities for crop improvement but, at the same time, provoked public concerns about its potential effect on human health and environment. Hence, commercialization of genetically modified crops (GMOs) is under strict control and limited by lengthy and expensive regulatory assessment procedures [260]. 

The advanced GE technology assists to produce precise and targeted mutations without the integration of any DNA sequence in plant genomes. This permits the development of non-transgenic crops with increased yield and improved quality and stress tolerance [261]. These approaches are speedy in contrast to traditional breeding techniques and allow development of transgene-free plants [156]. Plants produced via GE approaches are very similar to plants developed by conventional breeding. Additionally, GE technology takes less time to incorporate desired traits into the plant genome as compared to transgenic breeding, mutation breeding, and traditional breeding. It will take approximately 4–6 years to develop a GE plant with desired trait as compared to the transgenic breeding, which require 8–12 years for the creation of transgenic plant. On the other hand, mutation breeding and conventional breeding need 8–10 years approximately to obtain a desired phenotype [258]. The emergence of advanced GE tools not only revolutionized the world of science, but its economics are also very spectacular as compared to genetically engineered plants. Generally, the total expenditure required to execute a single transformation event would have been approximately a quarter of a million US dollars [262], while the budget needed for GE could be $30 [263], which is astonishingly economical and reliable. Consequently, large amounts of money can be saved on developing and approving genome-edited crops, avoiding laborious and time-consuming field experiments which normally demand many years to regulate a GM crop. In addition, it will eliminate the uncertainty and fear regarding the use of GM crops [212]

There is a primary need to review the current rules and regulations regarding GMOs. In addition, genome manipulation done via GE tools are quite different from a transgenic approach. For example, mutations produced by CIRSPR/Cas9 are small indels as compared to large gene sequence insertion or deletion [264]. Such small indels are most often produced in plants under normal growth environments and can also be generated through conventional mutagens. Additionally, in contrast to GMOs which need stable integration of foreign DNA in the genome, CRISPR/Cas9-mediated GE can be used to develop DNA-free non-transgenic plants with improved traits. As far as the regulatory affairs of these gene-edited plants are concerned, there is no international regulatory framework present at the moment. Two major stakeholders, the USA and European Union (EU), have opposite policies for the regulation of genome-edited plants. The United States Department of Agriculture (USDA) has exempted GE crops from its strict rules and regulations [265], while the EU holds the position to treat genome-edited plants as GMOs. Recently, the European Court of Justice has ordered the verdict that GE plants should be subjected to similar regulatory procedures as in the case of GMOs [266]. This judicial ruling may impede investment in GE techniques and limit their use in modern plant breeding platforms in European countries. In Germany, CIBUSTM canola cases are undecided and no legitimate information has been issued by the European Commission (EC) [267]. In 2011, independent legal experts at the EU suggested some legal categorization of modern plant breeding approaches, including GE technology. A committee was formed by the EC, called the “New Techniques Working Group”, to evaluate plants developed using different breeding techniques that fall under the category of GMOs legislation. By the end of 2011, the assessment was completed and the report finalized but it was never published [268]. Although, some important regulatory entities of the EU (including the French High Council for Biotechnology, the European Plant Science Organization, German Academy of Sciences, and the British Biotechnology and Biological Sciences Research Council) have already proposed that the assessment of GE plants should be based on the specific trait improvement instead of technology executed to develop them. However, a study was carried out by the German Federal Agency for Nature Conservation about GE organisms and they decided that GE organisms must be treated under the same regulations as GMOs, arguing that since GE is a strategy to manipulate the genome to produce targeted modification linked with unfamiliar risks, regardless of genome alterations that happen in nature [269].

In several countries, emerging crop editing tools like meganulcease, TALENs, ZFNs, and CRISPR/Cas9 have been applied for the past decade and they do not come under the category of GMO regulatory laws. The USA and Canada regard gene editing as equivalent to traditional breeding. The United States Department of Agriculture (USDA) granted permission to regularize and develop the CRISPR/Cas9-mediated genome-edited crops. Besides the USA, many other countries such as Brazil, Chile, and Argentina have established advanced regulatory principles for genome-edited crops. Every new discovery in biotechnology has been flawlessly approved due to the trait-based scheme in Canada. A strong and authentic regulatory policy is required to distinguish between GMOs and GE plants. Unfortunately, many countries have not established a clear regulatory policy for GE plants. The extensive use of GE strategies brings many challenges for regulatory bodies, as it requires great technical expertise and reliable evaluating procedures for the regulation of GE crops. Evidently, science-based guidelines that judge genome edited plants in a similar way as plants developed by conventional breeding programs are required to boost the applications of GE for crop improvement. For this, many countries such as the United States, Argentina, Australia, Brazil, Canada, and Chile have issued legal interpretations of various omissions in regulatory rules and exempted GE crops from the strict regulations of GMOs. However, this exemption may be dependent on some strict requirements like absence of foreign gene (Australia), no signs of pest characteristics (USA), and type of trait modification (Canada and other countries). 

The USA is the main stakeholder in the world and several regulatory authorities govern the regulation of GE crops including the Food and Drug Administration (FDA), Environmental Protection Agency (EPA), Animal and Plant Health Inspection Service (APHIS), and the and United States Department of Agriculture (USDA). The current policy of the USA regarding GE crops was developed by the USDA and depends on the “Plant Protection Act”. Any GE plant that poses pest characteristics and food safety issues is closely assessed and monitored by the regulatory bodies (USDA, EPA, and FDA). The USDA does not treat GE plants under GMOs regulations (https://www.aphis.usda.gov/aphis/ourfocus/biotechnology/brs-news-and-information/pbi-details). The APHIS has proposed many verdicts regarding the risk assessment of DNA-free GE crops and suggested modifications to the rules in order to eradicate the legislation application about pest and GE crops in 2017 [270]. The United States Department of Agriculture (USDA) acknowledged gene editing as a much faster form of traditional breeding. The USDA has allowed more than ten case-by-case studies of genome editing for cultivation without regulatory permits. These included the development of a high level of amylopectin producing Wax corn by applying the CRISPR/Cas9 tool which has been mutated for the *Wx1* gene. Similarly, a CRISPR/Cas9 strategy was applied to produce browning resistance to white button mushrooms by mutating the *polyphenol oxidase* gene at the Pennsylvania State University [265]. Herbicide-resistant rape seed was produced by the RTDS mechanism. In soybean, drought-resistant genes such as *Drb2b* and *Drb2a* were knocked out using the CRIPSR/Cas9 system. *Setaria viridis* was subjected to the CRISPR/Cas9 technique for delayed flowering by disrupting the *ID1* gene and the *Camelina* genome was edited by Yield10 Bioscience for enhanced oil production. In addition, low phytate level corn has been established using Dow’s ZFN, and resistant wheat against powdery mildew, soybean with a mutated *FAD3* gene, and potato with *PPO* knockout using a TALENs approach were also approved.

Similarly, there is no difference in Canada’s approach towards the GE techniques from the techniques that have foreshadowed it. Canadian plants with novel traits (PNTs) regulations are activated only if the technique produces any specific trait, causing toxicity, allergenicity, and effects on any other organism. All the plant cultivars with specific traits are subjected to PNT regulations, irrespective of how they were produced, suggesting that the plant cultivar could be produced via conventional breeding, conventional mutagenesis, genetic engineering or gene editing. It is anticipated that some of GE techniques may produce novel cultivars that are PNTs, while many of them may not be treated under the PNTs regulations. Thus, in Canada, plant cultivars that are carried through the PNT regulations need open release approval from Health Canada and the Canadian Food Inspection Agency (CFIA) in order to register as approved cultivars for commercial use by industry [271].

Argentina established a functional regulatory framework for regularization of modern plant breeding products (Whelan 2015) [272]. Policy-makers and regulatory bodies have made flexible assessment protocols that depend on case-by-case evaluations. Fundamentally, the regulatory framework of Argentina determines the overall process of developing a GE plant. The plant developed without any transgene integration has been designated as non-GMO. Moreover, if any transgene strategy was applied but the final product is DNA-free, then this is also treated as non-GMO. A regulating body in Argentina, CONABIA, assesses the genome-edited material before giving approval. Under the rule No. 763/11, a simple deletion in genome is not regarded as a GM crop.

Most of the GE tools are introduced by industries, but the CRISPR/Cas technology was discovered by academic research groups. These academic institutions and different companies are contesting to establish intellectual property (IP) sets for speedy commercialization of CRISPR/Cas-based products. Since 2005, a 15 fold increase has been reported in the number of patent applications and 42 patent applications were registered in the USA in 2014. Over the last couple of years, investment in GE bio-enterprise has increased fivefold [273]. It was estimated that the market value of GE technology was about $1.84 billion at the end of 2014, and it is predicted to grow with a 13.75% compound annual growth rate of about $3.51 billion by 2019 [274]. Additionally, the private companies that use CRISPR/Cas in the sector of agriculture, health, and industry have equally played a significant part in the current growth of the GE market. Over $600 million has been received by major companies which use CRISPR/Cas technology over the last decade [275].

To conclude, regulatory authorities need to develop comprehensive regulatory frameworks which direct the utilization of GE tools without constraining research. Furthermore, issues of IP rights and licensing policies need to be scrutinized for GE plants which can be used for commercial purposes. 

## 8. Conclusion and Outlook

The production of safe, low-cost, and nutritive food by adopting sustainable agricultural practices will be a huge task. In this regard, the availability of modern technologies to improve cultivars will be a vital aspect. GE is a powerful tool which is expected to play a crucial role in meeting the increasing demands of crop production to fulfill the needs of an exploding population under a climate change scenario. As compared to conventional breeding methodologies, the molecular breeding strategies aided by GE tools allow scientists to precisely target and edit for desired traits. GE can be used to enhance crop productivity, nutritional value, and develop resistance against biotic as well as abiotic stressors by improving the crop genome. The advanced tools in plant GE have been extensively employed to edit crops for a specific agronomic trait and have been utilized in several breeding platforms for carrying the desired trait for the development of an elite local variety. Thus, modern plant breeding approaches will increase the performance of plant breeding, and gene-edited elite cultivars can be approved for cultivation in specified locations without strict regulatory laws. For the last two decades SSNs such as meganucleases, ZFNs, and TALENs have revolutionized plant GE. These SSNs have many applications in plant GE and can be used for gene insertion, gene deletion, and increasing the efficiency of homologous recombination which allows for more precise and accurate events of gene replacement. 

Beside other GE techniques, CRISPR/Cas9 is the most powerful tool for crop improvement. In many plant systems it has been vigorously applied over the last five years for combating abiotic and biotic stressors and to improve other agronomic traits. CRISPR/Cas9 as a GE technology for site-direct mutagenesis has many excellent characteristics including great target specificity, easiness to execute, and low cost, which are unachievable through conventional mutagenic strategies. Further, CRISPR/Cas9 is superior to other first-generation SSNs because RNAs guide the Cas9 nuclease instead of proteins. Several Cas9-mediated techniques are being employed in different plant varieties, and these techniques will offer exceptional knowledge about plant biology and facilitate us to develop improved cultivars with great accuracy and speed via modern plant breeding. Recently some striking developments have been achieved in the CRISPR/Cas9 toolbox to increase the targeted mutagenesis with increased efficiency via base editing, multiplex GE [276], and generation of DNA-free plants. The CRSIPR/Cas9 is a versatile tool for plant GE, due to the fact of its sophisticated toolbox of Cas9 variants such as the CRISPR/Cpf1 system and online accessible bioinformatics tools for designing highly precise delivery systems. The CRISPR/Cas9-based precise GE produces gene replacement, gene insertion, and knockout mutations that are rapidly being used to increase yield, improve quality, and enhance tolerance in crops to boost crop domestication and hybrid breeding. Moreover, CRISPR/Cas9 technology is gaining interest day by day and will be a fundamental GE approach to developing improved plants with desired traits that will aid in accomplishing the goal of zero hunger in the world. 

Although CRISPR/Cas9-mediated GE has gained remarkable achievements in crop improvement, there are certain challenges that need to be addressed to develop a more efficient system for plant GE. This includes assembling pangenomes for crop improvement, programmed identification of candidate sites for gene editing via functional genomics, designing of highly efficient delivery systems for GE, and reducing the frequency of off-target editing, deciphering novel pathways for this reduction, and optimization of the Cas9 function. The major pitfalls of CRISPR/Cas9 is the inefficient delivery system for plant transformation because the current protocols are limited to certain tissues [277], genotypes, and crop varieties. The packaging of Cas proteins into delivery vectors poses large barriers for efficient delivery of CRISPR/Cas machinery. Recently, some novel cargo-vector systems have been introduced which show promising potential for efficient delivery systems. For example, carbon nanotubes have been utilized to transfer CRISPR/Cas9 editing constructs into plant leaves. Some other nano-products such as mesoporous silica nanoparticles and layered double hydroxides also have great potential to broaden the accessibility of delivery systems, as they have high transformation efficiencies and little toxicity and cellular damage. Developing improved delivery systems will be vital for efficient targeted and more precise GE for crop improvement. On the other hand, the frequency of off-target effects needs to be addressed more comprehensively, as there are many safety issues linked with CRISRP/Cas9-based bio-products. Luckily, off-target mutations are mostly bearable in plants and mutants, and off-target effects can be detected and eliminated through segregation over successive crosses. Selection of Cas9 requiring long PAMs and designing of sgRNA with close affinity for target sequence, may help to reduce the off-target effects in the future. Therefore, continuous efforts are required to overcome these hurdles in order to increase the experimental versatility and applied skills of the CRISPR/Cas9 toolbox in the future. 

The advancement in modern breeding approaches has been greatly acknowledged as an innovation in our capacity to manipulate genomes and has subsequently challenged our understanding and assessment of current regulatory policies. As GE tools are extensively employed in plants, the safety of GE plants is the matter of debate around the globe. Development of regulatory policies for novel crop innovations should be multidimensional, transparent, and be able to distinguish between GMOs and GE events. Hence, to explore the large prospective of modern plant breeding approaches for improved yield and food security, it is necessary to illuminate the clear status of these approaches, including GE, and to fix current regulatory uncertainties. Harnessing the innovative ideas of system biology, synthetic biology, next-generation sequencing, and the latest developments in functional genomic approaches integrated with the advanced tools of CRISPR/Cas9 will permit the development of smart crops with higher yields and improved qualities. In the near future, CRISPR/Cas9 technology can be integrated with speed breeding programs to revolutionize the global agriculture and promise of food security.

## Figures and Tables

**Table 1:** Applications of first-generation genome editing tools in crop plants.

Gene Editor Nucleases	Species	Modification Type	Delivery Technique	Repair Pathway	Target Gene	Desired Trait	Reference
Meganuclease	*Zea mays*	Gene knockout	*Agrobacterium*-mediated transformation	NHEJ	*MS26*	Male-sterile plants	[50]
	*Gossypium hirsutum*	Trait stacking	Particle bombardment	HR	*EPSPS*	Herbicide tolerance	[30]
	*Zea mays*	Gene knockout	*Agrobacterium*-mediated transformation	HR	*LG1*	Heritable targeted mutagenesis	[51]
Zinc-finger nucleases	*Oryza sativa*	Trait stacking	*Agrobacterium*-mediated transformation	HR	*OsQQR*	Detection of safe harbor loci	[37]
	*Zea mays*	Trait stacking	Microparticle bombardment	HR	*ZmTLP*	Herbicide tolerance	[52]
	*Brassica napus*	Gene expression	*Agrobacterium*-mediated transformation	Transcriptional activation	*KasII*	Improved seed oil composition	[21]
	*Glycine max*	Gene knockout	*Agrobacterium rhizogenes*	HR	*DCL*	Heritable transmission	[40]
	*Zea mays*	Gene replacement	Whiskers	NHEJ	*IPK1*	Herbicide tolerance	[39]
TALENs	*Saccharum officinarum*	Gene knockout	*Agrobacterium*-mediated transformation	NHEJ	*COMT*	Improved saccharification efficiency	[53]
	*Zea mays*	Frame-shift mutation	*Agrobacterium*-mediated transformation	NHEJ	*ZmMTL*	Induction of haploid plants	[54]
	*Glycine max*	Gene knockout	*Agrobacterium rhizogenes*	NHEJ	*GmPDS11, GmPDS18*	Albino and dwarf phenotype	[55]
	*Glycine max*	Gene stacking	*Agrobacterium rhizogenes*	NHEJ	*FAD2-1A, FAD2-1B, FAD3A*	High oleic, low linoleic contents	[56]
	*Solanum tuberosum*	Gene knockout	Particle bombardment	NHEJ	*VInv*	Minimizing reducing sugars	[57]
	*Saccharum officinarum*	Gene knockout	*Agrobacterium*-mediated transformation	NHEJ	*COMT*	Improved cell wall composition	[58]
	*Solanum tuberosum*	Gene knockout	Particle bombardment	NHEJ	*ALS*	Transient expression in protoplasts	[59]
	*Zea mays*	Gene knockout	*Agrobacterium*-mediated transformation	NHEJ	*ZmGL2*	Reduced epicuticular wax in leaves	[60]
	*Oryza sativa*	Gene knockout	*Agrobacterium*-mediated transformation	NHEJ	*OsBADH2*	Fragrant rice	[61]
	*Triticum aestivum*	Gene knockout	*Agrobacterium*-mediated transformation	NHEJ	*MLO*	Powdery mildew resistance	[62]
	*Hordeum vulgare*	Gene knockout	*Agrobacterium*-mediated transformation	NHEJ	Transgene	*GFP*	[63]
	*Glycine max*	Gene knockout	*Agrobacterium rhizogenes*	NHEJ	*FAD2-1A/B*	Improved oil quality	[64]
	*Nicotiana tabacum*	Gene knockout	Peg-mediated protoplast transformation	NHEJ	*Sur A, Sur B*	Targeted mutation	[42]
	*Oryza sativa*	Gene knockout	*Agrobacterium*-mediated transformation	NHEJ	*Os11N3*	Bacterial blight resistance	[43]

**Description:** Non-homologous end joining (NHEJ), Homology repair (HR), Male sterile 26 (*MS26*), 3-phosphoshikimate 1-carboxyvinyltransferase 2 (*EPSPS*), Liguleless 1 (*LG1*), Trait landing pads (*ZmTLP*), 3-oxoacyl-[acyl-carrier-protein] synthase II, chloroplastic (*KasII*), Protein DCL homolog, chloroplastic (*DCL*), Inositol pentakisphosphate 2-kinase (*IPK1*), Catechol-O-methyltransferase (*COMT*), MATRILINEAL (*MTL*), Phytoene desaturase (*PDS*), Fatty acid desaturase (*FAD*), Vacuolar invertase gene (*VInv*), Acetolactate synthase gene (*ALS*), Maize glossy2 (*GL2*), Betaine aldehyde dehydrogenase (*BADH2*), MILDEW-RESISTANCE LOCU (*MLO*), Green fluorescent protein (*GFP*), Acetolactate synthase genes (*Sur A, Sur B*), Rice bacterial blight susceptibility gene (*Os11N3).*

**Table 2:** List of various single guide RNA (sgRNA) designing bioinformatics tools for the CRISPR/Cas9 system.

Tool Name	Description & Function	Year	Web Link	Reference
CRISPRlnc	Design sgRNA for lncRNAs, works for all species	2019	(http://www.crisprlnc.org)	[105]
CRISPR-Local	Design sgRNA for non-reference cultivars, predict sgRNA that can target multiple genes	2018	(http://crispr.hzau.edu.cn/CRISPR-Local/)	[106]
sgRNA Scorer 2.0	Design sgRNA for several PAM sites	2017	(http://crispr.med.harvard.edu/sgRNAScorerV2)	[107]
CRISPR-P 2.0	Predict on-target scores, analyze and detect guide sequence	2017	(http://cbi.hzau.edu.cn/CRISPR2/)	[108]
CRISPRpred	Efficient designing of sgRNA based on target in silico prediction	2017	(https://github.com/khaled-buet/CRISPRpred)	[109]
CRISPR-DO	Specific for both coding and non-coding targets, provides information regarding off-targeted sites and its functional conservation	2016	(http://cistrome.org/crispr/)	[110]
phytoCRISP-Ex	UNIX-based standalone, Cas9 target prediction	2016	(http://www.phytocrispex.biologie.ens.fr/CRISP-Ex/)	[111]
CRISPy	Target prediction for sgRNA, graphical representation of results	2016	(http://crispy.secondarymetabolites.org/)	[112]
Cas-Designer	RNA-guided endonucleases, provides all information about off-targets and out-of frame scores	2015	(http://rgenome.net/cas-designer/)	[113]
CCTop	Predict target sgRNA sequence based on possible off-targets	2015	(https://crispr.cos.uni-heidelberg.de/)	[100]
Azimuth	Design sgRNA for both on-target and off-target models	2015	(https://research.microsoft.com/en-us/projects/azimuth/)	[114]
CRISPRdirect	Design sgRNA with minimal off-targets	2014	(https://crispr.dbcls.jp/)	[115]
CRISPR-PLANT	Construct specific sgRNAs for particular plant species	2014	(https://www.genome.arizona.edu/crispr/)	[102]
CRISPRseek	Screen sgRNA for targeted sequences, produce cleavage scores for predicted off-targets	2014	(https://www.bioconductor.org/packages/release/bioc/html/CRISPRseek.html)	[116]
Cas-OFFinder	Based on RNA-guided endonucleases, robust for detecting off-target sites	2014	(http://www.rgenome.net/cas-oinder/)	[117]
E-CRISP	Potential target site evaluation	2014	(https://www.e-crisp.org/E-CRISP/designcrispr.html)	[118]
SSFinder	High-throughput detection of target sites	2014	(https://code.google.com/p/ssinder/)	[119]
GPP Web Portal	Produce potential sgRNA scores	2014	(https://www.broadinstitute.org/rnai/public/analysis-tools/sgrnadesign)	[120]
CRISPR-P	Generate synthetic sgRNA, predict potential sites for enzyme cut	2014	(https://cbi.hzau.edu.cn/crispr)	[104]
CHOPCHOP	Detect optimal target sites for sgRNA, produce potential scores for target sites	2014	(https://chopchop.cbu.uib.no/)	[121]
sgRNAcas9	Rapid design of sgRNA with less off-targets	2014	(https://www.biootools.com/col.jsp?id=103/)	[122]
CRISPR Design	Precise sgRNA construction for target sites, assess off-target sites	2013	(http://www.genome-engineering.org)	[101]

**Table 3:** Summary of disease-resistant crops developed via CRISPR/Cas9.

Crop	Target Gene	Pathogen	Gene Function	Trait Improvement	Editing Result	Repair Pathway	Delivery Technique	Reference
*Oryza sativa*	*eIF4G*	*Rice tungro spherical virus*	Translation initiation factor	Resistance against *Rice tungro spherical virus*	Knock-out	NHEJ	*Agrobacterium*-mediated transformation	[215]
*Vitis vinifera*	*VvWRKY52*	*Botrytis cinerea*	Transcription factor	Increased resistance against *Botrytis cinerea*	Knock-out	NHEJ	*Agrobacterium*-mediated transformation	[216]
*Gossypium hirsutum*	*Gh14-3-3d*	*Verticillium dahliae*	Negative regulator of disease resistance	Resistance to Cotton verticillium wilt	Knock-in	NHEJ	*Agrobacterium*-mediated transformation	[217]
*Solanum lycopersicum*	*SlJAZ2*	*Pseudomonas syringae*	co-receptor of coronatine	Bacterial speck resistant	Knock-out	NHEJ	*Agrobacterium*-mediated transformation	[218]
*Solanum lycopersicum*	CP and Rep sequences	*Tomato yellow leaf curl virus*	Negative regulator of viral resistance	Improved resistance against *Tomato yellow leaf curl virus*	Knock-out	NHEJ	*Agrobacterium*-mediated transformation	[219]
*Solanum lycopersicum*	*SlMlo1*	*Oidium neolycopersici*	Encoding powdery mildew resistance	Improved resistant against powdery mildew	Knock-out	NHEJ	*Agrobacterium*-mediated transformation	[209]
*Triticum aestivum*	*EDR1*	*Erysiphe cichoracearum*	Encoding powdery mildew resistance	Improved resistant against powdery mildew	Knock-out	NHEJ	Particle bombardment	[208]
*Citrus paradise*	*CsLOB1*	*Xanthomonas citri* subsp. *citri*	Increase susceptibility against citrus canker	Citrus canker resistant	Knock-out	NHEJ	*Agrobacterium*-mediated transformation	[207]
*Citrus sinensis*	CsLOB1	*Xanthomonas citri* subsp. *citri*	Increase susceptibility against citrus canker	Citrus canker resistant	Knock-out	NHEJ	*Agrobacterium*-mediated transformation	[206]
*Oryza sativa*	*OsERF922*	*Magnaporthe oryzae*	*ERF* transcription factor	Resistance against blast fungus	Knock-out	NHEJ	*Agrobacterium*-mediated transformation	[204]
*Cucumis sativus*	*eIF4E*	Multiple viruses	Translation initiation factor	Broad virus resistance	Knock-out	NHEJ	*Agrobacterium*-mediated transformation	[220]
*Oryza sativa*	*OsSWEET13*	*X. oryzae* pv. *oryzae*	Sucrose transporter gene	Resistance against bacterial blight	Knock-out	NHEJ	*Agrobacterium*-mediated transformation	[205]

**Description:** Translation initiation factor 4 gamma gene (*eIF4G*), WRKY transcription factor 52 (*WRKY52*), 14-3-3 protein 6-like (*Gh14-3-3d*), Jasmonate ZIM-domain protein 2 (*JAZ2*), Powdery mildew resistance protein (*Mlo1*), Enhanced disease resistance1 (*EDR1*), LATERAL ORGAN BOUNDARIES 1 (*CsLOB1*), Ethylene-responsive gene (*OsERF922*), Eukaryotic translation initiation factor 4E-1 (*elF4E*), Bidirectional sugar transporter SWEET13-like (*SWEET13*).

**Table 4:** Summary of CRISPR/Cas9 applications in major crops for abiotic stress tolerance.

Crop	Target Gene	Trait Study	Editing Result	Repair Mechanism	Delivery Technique	Reference
*Oryza sativa*	*OsNAC041*	Salinity tolerance	Knockout	NHEJ	*Agrobacterium*-mediated transformation	[228]
*Oryza sativa*	*OsOTS1*	Salinity tolerance	Knockout	NHEJ	*Agrobacterium*-mediated transformation	[229]
*Oryza sativa*	*OsRR22*	Salinity tolerance	Knockout	NHEJ	*Agrobacterium*-mediated transformation	[230]
*Solanum lycopersicum*	*SlNPR1*	Drought tolerance	Knockout	NHEJ	*Agrobacterium*-mediated transformation	[231]
*Glycine max*	*Drb2a, Drb2b*	Drought and salt tolerance	Knockout	NHEJ	*Agrobacterium rhizogenes*	[225]
*Oryza sativa*	*OsNAC14*	Drought tolerance	Knock-in	HDR	*Agrobacterium*-mediated transformation	[232]
*Oryza sativa*	*SAPK1* and *SAPK2*	Salinity tolerance	Knockout	NHEJ	*Agrobacterium*-mediated transformation	[233]
*Zea mays*	*ZmHKT1*	Salinity tolerance	Knockout	NHEJ	*Agrobacterium*-mediated transformation	[234]
*Solanum lycopersicum*	*SlCBF1*	Cold tolerance	Knockout	NHEJ	*Agrobacterium*-mediated transformation	[235]
*Triticum aestivum*	*TaDREB2, TaDREB3*	Drought tolerance	Knockout	NHEJ	PEG-mediated transformation	[221]
*Oryza sativa*	*OsAnn3*	Cold tolerance	Knockout	NHEJ	*Agrobacterium*-mediated transformation	[222]
*Oryza sativa*	*SAPK2*	Drought and salinity tolerance	Knockout	NHEJ	*Agrobacterium*-mediated transformation	[223]
*Zea mays*	*ARGOS8*	Drought tolerance	Knockout	HDR	Particle bombardment	[224]
*Solanum lycopersicum*	*SlMAPK3*	Drought tolerance	Knockout	NHEJ	*Agrobacterium*-mediated transformation	[226]
*Oryza sativa*	*OsMPK2, OsPDS, OsBADH2*	Multiple stress tolerance	Knockout	HDR	Particle bombardment	[26]

**Description:** NAC transcription factor coding gene (*OsNAC041*), Small Ubiquitin-like Modifier (*OsOTS1*), Two-component response regulator (*OsRR22*), Regulatory protein NPR1 (*SlNPR1*), dsRNA-binding protein (*Drb*), NAC domain-containing protein 2 (*OsNAC14*), ABA-activated protein kinase 1 (*SAPK1*), Sodium transporter HKT1 (*ZmHKT1*), C-repeat-binding factor-1 (*SlCBF1*), Wheat dehydration responsive element binding protein 2 (*TaDREB2*), Wheat ethylene responsive factor 3 (*TaDREB3*), Aluminum-induced protein superfamily pseudogene (*ARGOS8*), Mitogen-activated protein kinase 3 (*SlMAPK3*), Mitogen-activated protein kinase 2 (*OsMPK2*).

**Table 5:** Summary of CRISPR/Cas9 applications in major crops for yield and quality improvement.

Crop	Target Gene	Trait Improvement	Editing	Repair Mechanism	Delivery Technique	Reference
*Triticum aestivum*	*TaGW2*	Grain weight	Knockout	HR	Particle bombardment	[236]
*Oryza sativa*	*OsAAP3*	Grain yield	Knock-in	NHEJ	*Agrobacterium*-mediated transformation	[237]
*Oryza sativa*	*OsCCD7*	High-tillering	Knockout	NHEJ	*Agrobacterium*-mediated transformation	[252]
*Glycine max*	*GmFT2a*	Delayed flowering	Knockout	NHEJ	*Agrobacterium*-mediated transformation	[253]
*Oryza sativa*	*GW5*	Grain weight	Knockout	NHEJ	*Agrobacterium*-mediated transformation	[238]
*Oryza sativa*	*Hd2*, *Hd 4*, *Hd5*	Early heading	Knockout	NHEJ	*Agrobacterium*-mediated transformation	[242]
*Oryza sativa*	*OsSWEET11*	Grain weight	Knockout	NHEJ	*Agrobacterium*-mediated transformation	[243]
*Solanum lycopersicum*	*SP5G*	Early yielding	Knockout	NHEJ	*Agrobacterium*-mediated transformation	[254]
*Oryza sativa*	*OsGRF4*	Grain size	Knock-in	NHEJ	*Agrobacterium*-mediated transformation	[239]
*Oryza sativa*	*IPA, GS3, DEP1, Gn1a*	Improved yield	Knockout	NHEJ	*Agrobacterium*-mediated transformation	[240]
*Oryza sativa*	*GS3*, *GW2*, *GW5*, *TGW6*	Grain weight	Knockout	NHEJ	*Agrobacterium*-mediated transformation	[241]
*Triticum aestivum*	*GASR7*	Kernel weight	Knockout	HDR	Particle bombardment	[148]
*Triticum aestivum*	*α–gliadin*	Low gluten	Knockout	HDR	Particle bombardment	[247]
*Oryza sativa*	*Waxy*	Enhanced glutinosity	Knockout	NHEJ	*Agrobacterium*-mediated transformation	[245]
*Solanum lycopersicum*	*lncRNA1459*	long shelf life	Knockout	NHEJ	*Agrobacterium*-mediated transformation	[250]
*Solanum lycopersicum*	*SGR1, LCY-E, Blc, LCY-B1*	Increased lycopene	Knockout	NHEJ	*Agrobacterium*-mediated transformation	[251]
*Oryza sativa*	*SBEIIb*	Amylose, starch resistance	Knockout	NHEJ	*Agrobacterium*-mediated transformation	[246]
*Glycine max*	*FAD2-1A, FAD2-1B*	Improved oil quality	Knockout	NHEJ	*Agrobacterium*-mediated transformation	[163]
*Solanum tuberosum*	*GBSS*	Increase amylopectin/amylose	Knockout	NHEJ	PEG-mediated transfection	[152]
*Solanum lycopersicum*	*SlGAD2, SlGAD3*	Enhance Υ-Aminobutyric acid	Knockout	NHEJ	*Agrobacterium*-mediated transformation	[255]
*Zea mays*	*PPR*, *RPL*	Reduced zein protein	Knockout	NHEJ	*Agrobacterium*-mediated transformation	[182]

**Description:** Carotenoid cleavage dioxygenase 7, chloroplastic-like (*CCD7*), Phosphatidylethanolamine-binding protein FT2a (*FT2a*), Protein IQ-DOMAIN 14 (*GW5*), Protein SELF PRUNING 5G (*SP5G*), Growth-regulating factor 4-like (*GRF4*), Glutathione synthetase (GS3), Protein STRICTOSIDINE SYNTHASE-LIKE 10 (*TGW6*), Cytokinin dehydrogenase 2-like (*Gn1a*), Keratin-associated protein 5-5 (*DEP1*), Ubiquitin-protein ligase (*GW2*), GA-induced protein (*GASR7*), Senescence-inducible chloroplast stay-green protein 1 (*SGR1*), Lycopene epsilon-cyclase (*LCY-E*), Glycoside hydrolase family 13 protein (*SBEIIb*), Granule-bound starch synthase (*GBSS*), 2-oxoglutarate-dependent dioxygenase 2 (*GAD2*), Pentatricopeptide repeat (*PPR*), Ribosomal protein lateral (*RPL*).

## Abbreviations

CRISPR	Clustered regularly interspaced short palindromic repeats
Cas9	CRISPR-associated protein 9
GE	Genome Editing
SSNs	Site-specific nucleases
DSB	Double-stranded breaks
NHEJ	Non-homologous end joining
HDR	Homology-directed recombination
MNs	Meganulceases
ZFNs	Zinc-finger nucleases
TALENs	Transcription activator-like effector nucleases
TALEs	Transcription activator like effectors
RVD	Repeat variable di-residues
sgRNA	single guide RNA
Pre-crRNA	Precursor CRISPR-RNA
PAM	Protospacer adjacent motif
RNPs	Ribonucleoproteins
GMO	Genetically modified organism
CBE	Cytosine base editor
ABE	Adenine base editor
